# Vertebral-specific activation of the CX3CL1/ICAM-1 signaling network mediates non-small-cell lung cancer spinal metastasis by engaging tumor cell-vertebral bone marrow endothelial cell interactions

**DOI:** 10.7150/thno.54235

**Published:** 2021-03-04

**Authors:** Ketao Wang, Libo Jiang, Annan Hu, Chi Sun, Lei Zhou, Yiwei Huang, Qing Chen, Jian Dong, Xiaogang Zhou, Feng Zhang

**Affiliations:** 1 Department of Orthopaedic Surgery, Zhongshan Hospital, Fudan University, Shanghai 200032, China.; 2 JMS Burn and Reconstruction Center, Jackson, Mississippi, USA.; 3 Department of Orthopaedic Surgery, Zhongshan Hospital Wusong Branch, Fudan University, Shanghai 200940, China.; 4 Department of Thoracic Surgery, Zhongshan Hospital, Fudan University, Shanghai 200032, China.

**Keywords:** Non-small cell lung cancer, spinal metastasis, CX3CL1, ICAM-1, extravasation.

## Abstract

**Rationale:** The spine is one of the most common metastatic sites of non-small cell lung cancer (NSCLC), and NSCLC spinal metastasis results in serious consequences. Metastatic extravasation of disseminated cancer cells including increased invasiveness, adhesion and transendothelial migration is crucial for tumor metastasis. This study aimed to investigate the mechanisms underlying NSCLC spinal metastasis based on the C-X3-C motif chemokine ligand 1- (CX3CL1) and intercellular adhesion molecule-1- (ICAM-1) mediated signaling network.

**Methods:** Immunohistochemistry, western blotting, and reverse transcription-quantitative PCR were conducted to detect the distribution of CX3CL1/ICAM-1 in different organs. Transwell, adhesion, and transendothelial migration assays were performed to evaluate the regulatory effects of CX3CL1/ICAM-1 on NSCLC cell invasion, adhesion, and transendothelial migration *in vitro*. A spontaneous spinal metastasis mouse model was established via injection of NSCLC cells into the left cardiac ventricle of NOD/SCID mice. The effects of CX3CL1/ICAM-1 on NSCLC spinal metastasis *in vivo* were validated using bioluminescent, micro-computerized tomography, immunohistochemistry and histological analyses.

**Results:** CX3CL1 expression was specifically higher in vertebral bone compared with limb bones and lung tissue, and was associated with NSCLC spinal metastasis. Mechanically, vertebral bone marrow endothelial cells (VBMECs) enhanced NSCLC cell invasion via CX3CL1 signaling-mediated activation of the PI3K/AKT pathway. Furthermore, we found that VBMECs effectively induced ICAM-1-dependent NSCLC cell adhesion in coordination with platelets through the CX3CL1/ICAM-1/LFA-1 pathway. Meanwhile, CX3CL1 enhanced NSCLC cell transendothelial migration by increasing permeability of VBMECs via ICAM-1-dependent activation of the Src/GEF-H1 pathway. Interestingly, NSCLC cells were indicated to promote CX3CL1 secretion of VBMECs through MAPK14/ADMA17-dependent CX3CL1 release and NF-κB-dependent CX3CL1 synthesis. Based on these findings, we revealed a novel feedback cycle between circulating NSCLC cells and VBMECs mediated by CX3CL1/ICAM-1 signaling. Further disengagement of the CX3CL1/ICAM-1-mediated feedback cycle* in vivo* significantly restricted metastasis and prolonged mouse survival.

**Conclusions:** Our results indicated a unique feedback cycle between circulating NSCLC cells and VBMECs mediated by CX3CL1/ICAM-1 signaling, which is necessary for NSCLC spinal metastasis. This work provides a new perspective for underlying the mechanisms of NSCLC spinal metastasis and indicates potential novel targets for the prevention of NSCLC spinal metastasis.

## Introduction

Lung cancer is the second most common cancer and the leading cause of cancer-related deaths worldwide in both men and women 1. Non-small-cell lung cancer (NSCLC) is the most common type of lung cancer and accounts for more than 80% of all cases 2. Among late-stage NSCLC patients, more than 70% suffer from bone metastasis, 80% of which is located in the spine 3. When spinal metastasis occurs, it frequently results in bone destruction, pathological fracture, severe bone pain and neurological deficits. Although chemotherapy, radiotherapy, surgical procedures such as Enbloc resection and comprehensive treatment can reduce morbidity of NSCLC spinal metastasis, these treatments often do not significantly improve overall survival 4, 5. Therefore, there is an urgent need to investigate the mechanisms underlying NSCLC‑derived spinal metastasis and develop prevention or early treatment strategies for NSCLC spinal metastasis.

A series of complex biological processes are involved in tumor metastasis: the detachment of tumor cells from solid tumors; the entrance of shedding cells to the circulatory system and formation of circulatory tumor cells (CTCs); aggregation with other tumor cells, platelets or other cells to form CTC clusters; arrival at distant organs and penetrating local blood vessels; and *in situ* colonization and the related destruction of surrounding tissues 6, 7. These processes are dependent on several tumor‑regulated factors, including chemokines, cytokines, and adhesion molecules, that mediate angiogenesis, tumor cell survival, and invasion 7, 8.

The red bone marrow of vertebrae possesses a special structure of blood sinus, which contains numerous cytokines, enzymes, and hormones, especially chemokines 9. Chemokines are a family of small structurally related secreted cytokines that play crucial roles in inflammation and immunity 10. In recent years, chemokines have been shown to be involved in modulating various events of tumor progression, such as leukocyte recruitment, tumor cell migration, and proliferation 11. Among these chemokines, C‑X3‑C motif chemokine ligand 1 (CX3CL1) has been regarded as an essential mediator in tumor metastasis 12. Interestingly, CX3CL1 was found to be more abundant in the red bone marrow of vertebrae than in limb bone, which may account for tumor spinal metastasis 13, 14. Despite the possible role of CX3CL1 in tumor progression in spine, the effects of CX3CL1 on several aspects of NSCLC spinal metastasis, particularly on CTC extravasation to vertebral cancellous bone, which is the first and crucial step for tumor spinal metastasis, remain unknown.

Therefore, we hypothesized that increased expression of CX3CL1 in the spine facilitates circulating NSCLC cell adhesion and transendothelial migration, and initiates NSCLC spinal metastasis. To examine this hypothesis, we detected the content of CX3CL1 in vertebral cancellous bone and vertebral bone marrow endothelial cells (VBMECs), determined whether CX3CL1 influenced the biological behaviors of circulating NSCLC cells, such as adhesion, transendothelial migration, and invasion, and investigated the underlying mechanisms. Finally, we investigated whether disrupting CX3CL1-mediated signaling could restrict metastasis and prolong survival in intracardiac models *in vivo*. The results of the present study provide evidence that CX3CL1 facilitates circulating NSCLC cell extravasation to vertebral cancellous bone and highlight novel signaling pathways that warrant further study for the development of prevention or early treatment strategies for NSCLC spinal metastasis.

## Materials and Methods

### Clinical specimens and cell preparation

A total of 32 clinical specimens were obtained from the Department of Orthopedic Surgery and Department of Thoracic Surgery, Zhongshan Hospital, Fudan University (Shanghai, China) between December 2015 and August 2019. These included 11 cases suffering from primary NSCLC with spinal metastases (male/female, 7/4; age, 62 ± 7.6 years), 11 samples of primary NSCLC tumors (male/female, 6/5; age, 53 ± 6.2 years), five cases undergoing posterior lumbar interbody fusion (male/female, 4/1; age, 56 ± 8.4 years), and five samples suffering from osteoarthritis and undergone arthroplasty (male/female, 3/2; age, 64 ± 8.2 years). The present study was approved by the Ethics Committee of Zhongshan Hospital, Fudan University (approval no. Y2014‑185 and Y2019‑085), and informed consent was provided by all patients.

VBMECs and limb bone marrow endothelial cells (LBMECs) were isolated from fresh, healthy human vertebral cancellous bone from two of the five patients undergoing posterior lumbar interbody fusion and femoral cancellous bone from two of the five patients suffering arthroplasty, according to methods previously reported 14,15. BMECs were maintained in Dulbecco's modified Eagle medium (DMEM, Gibco, Grand Island, NY, USA) supplemented with low-serum growth supplement (Gibco) at 37 °C in an atmosphere of 5% CO_2_. A549 and H1975 cells were purchased from The Cell Bank of Type Culture Collection of the Chinese Academy of Sciences and maintained in DMEM supplemented with 10% FBS (Invitrogen Life Technologies) at 37 °C in an atmosphere of 5% CO_2_.

### Reagents and vectors

AKT inhibitor MK-2206 2HCI, PI3K inhibitor LY294002, MAPK14 inhibitor SB203580, and NF-κB inhibitor Bay11-7085 were purchased from Selleck Chemicals. The Src signaling pathway agonist tamoxifen was purchased from Sigma Aldrich. Lipofectamine^®^ 2000 was purchased from Invitrogen (cat. no. 11668019; Thermo Fisher Scientific, Waltham, MA, USA). Human *CX3CL1* and non-targeting small interfering RNAs (siRNAs) were purchased from Ribobio (Guangzhou, China) (*CX3CL1* siRNA: 5'‑GGACAAGCCACATAGGAAA‑3'). Human *ICAM-1* and non-targeting control (siRNAs) were purchased from GeneChem (Shanghai, China) (*ICAM-1* siRNA: 5'‑GCCCGAGCTCAAGTGTCTAAA3'). Human *ADAM17* and non-targeting control siRNAs were purchased from GeneChem. The siRNA sequence was as follows: 5'‑GAAGGTGAATCTAGCTTATTT‑3'. Human *CX3CR1*, lymphocyte function-associated antigen-1 (*LFA-1*), and non-targeting control small hairpin RNA (shRNA) lentivirus particles expressing a puromycin-resistant gene were purchased from Sigma-Aldrich (USA) (*CX3CR1* shRNA: 5'‑CCCTTCTGGACTCACTATTTG‑3'; *LFA-1* shRNA: 5'‑TTGAGGATGTCACCAATTAAC‑3). The ICAM-1- and LFA-1-overexpressing lentiviruses were purchased from GeneChem (Shanghai, China).

### Transfection

All plasmid transfections were performed using Lipofectamine^®^ 2000 Reagent (Thermo Fisher Scientific) according to the manufacturer's instructions. VBMECs were seeded into 6‑well plates until 70% confluence and incubated with control, *CX3CL1*, *ICAM-1*, or *ADAM17* siRNA mix at a final concentration of 100 nM, followed by transfection using Lipofectamine^®^ 2000 Reagent. A549 cells were transfected with scrambled control, *CX3CR1*, or *LFA-1* shRNA lentiviral particles. At 48-72 h after infection, cells were collected to evaluate knockdown (KD) efficiency using qPCR or western blot analyses, and the cells with highest knockdown efficiency were selected for subsequent experiments. For lentiviral infections, VBMECs, and H1975 and A549 cells (2 × 10^5^), were seeded onto 6-well plates followed by infection with control, ICAM-1-, or LFA-1-overexpressing lentiviruses in the presence of polybrene (6 µg/µl). Cells were sequentially incubated in puromycin (5 µg/mL) to establish stable shRNA cell lines.

### Western blot analysis

Total protein was extracted from tissues or cells using a lysis buffer with phenylmethanesulfonylfluoride (cat. no. ST505; Beyotime Institute of Biotechnology) containing a phosphorylase inhibitor (cat. no. 78445, Thermo Fisher Scientific). The protein concentration was determined with a bicinchoninic acid assay (Beyotime Institute of Biotechnology). A total of 40 µg of protein was separated by 10% SDS-PAGE, transferred to PVDF membrane (Millipore, Hayward, CA, USA), and blocked with 5% fat-free milk for 1 h at room temperature. The membranes were probed with primary antibodies overnight at 4 ºC followed by incubation with the appropriate secondary antibodies for 2 h at room temperature. Proteins were visualized using enhanced ECL substrate (cat. no. ab133406; Abcam). ImageJ 1.8.0 software (National Institutes of Health, Bethesda, MD, USA) was used to quantitatively compare the differences between groups. The antibodies used are shown in Table 1.

### Immunohistochemistry

Immunohistochemistry was performed as previously described 13. Briefly, specimens were fixed with 4% polyoxymethylene, embedded in paraffin, and sectioned at a thickness of 6 µm. Then, the sections were dewaxed in xylene and rehydrated in graded ethanol followed by incubation in H_2_O_2_ (3%) for 10 min to block endogenous peroxidase. Then, specimens were incubated with antibodies of interest at 4 °C overnight followed by incubation with the appropriate secondary antibodies for 45 min at room temperature. The sections were stained with 3,3'-diaminobenzidine, counterstained with hematoxylin, and observed under a microscope (Olympus‑IX51; Olympus Corporation, San Jose, CA, USA).

### Reverse transcription‑quantitative polymerase chain reaction (RT‑qPCR)

Total RNA was extracted from cells or collected from 20 µm thick sections of tissues using TRIzol reagent (Invitrogen) according to the manufacturer's instructions and reverse transcribed into cDNAs using the mRNA Selective PCR Kit (TaKaRa, Mountain View, CA, USA). PCR amplification was performed on the ABI 7900 96 HT series PCR machine (Applied Biosystems) using a SYBR PCR Master Mix Kit (Sigma-Aldrich, St. Louis, MO, USA). Reactions were performed at 95 °C for 3 min, followed by 40 cycles of 95 °C for 15 s and 60 °C for 30 s. The experiments were performed three times independently. Relative expression of cDNA was calculated by the 2^‑ΔΔCT^ method normalized to GAPDH. Sequences of the primers are provided in Table 2.

### Enzyme-linked immunosorbent assay (ELISA) analysis

The levels of CX3CL1 were detected using ELISA kits (Boster Bio, Wuhan, China) according to the manufacturer's instructions. The optical density (OD) was measured using a microplate reader at 450 nm (Bio-Rad Laboratories, Hercules, CA, USA).

### Cell co-culture and invasion

A 24-well Transwell chamber with 8 µm pores (cat. no. 3402; Corning, Corning, NY, USA) was used to detect cell invasion. The upper chambers were precoated with Matrigel (cat. no. E6909; Sigma‑Aldrich; Merck KGaA, Darmstadt, Germany). Following overnight serum starvation, control A549, shCX3CR1-A549, control H1975, or shCX3CR1-H1975 cells were seeded at a density of 20,000 cells into the upper chamber containing 100 µL conditioned culture media of control or CX3CL1-knockdown (CX3CL1-KD) VBMECs nourished with 10% FBS for 6 days. In addition, a culture system consisting of the indicated A549 or H1975 cells and VBMEC culture media was treated with the AKT inhibitor MK-2206 2HCI (3 µM) or the PI3K inhibitor LY294002 (20 µM). A total of 600 µL DMEM containing 10% FBS was added to the lower chamber. Following incubation for 36 h, the upper surfaces were gently swabbed and the lower membrane was fixed with 4% paraformaldehyde for 15 min and stained with 0.1% Crystal Violet for 15 min at room temperature. The number of cells was determined under a microscope. Assays were quantified by counting the number of stained cells in five randomly selected fields in each well.

### Adhesion assay

The parallel-plate flow chamber perfusion system (cat. no. C901, Naturethink, Shanghai, China) was used to simulate blood flow of the vertebral blood sinus 16, 17. Monolayer VBMECs were seeded at the bottom of the middle chamber as the fixed phase of the parallel-plate flow chamber, and tumor cells labeled with 2',7'-bis-(2-carboxyethyl)5-(and-6)-carboxyfluorescein acetoxymethyl ester (Fanbo, Beijing, China) at a density of 5 × 10^5^/mL with serum-free medium served as the mobile phase. Platelets (pl) at a concentration of 300 × 10^9^ pl/L were added into the flow. Cells were perfused for 2 min at wall shear rates of 1000 s^-1^ at the center of the channel (the blood analog was pushed through the parallel-plate flow chamber by syringe pump at a flow rate of 0.3 mL/min). After cells flowed across the fixed phase of endothelial cells, nonadherent tumor cells were washed away, and the number of adherent fluorescent tumor cells was quantitated under a fluorescence microscope (Olympus BX53).

### Transendothelial migration assay

A 24-well Transwell chamber with 8 µm pores (cat. no. 3402; Corning, Inc.) was used for the transendothelial migration assay. The upper chambers were precoated with Matrigel following incubation of CX3CL1-KD, ICAM-1-KD, CX3CL1-KD+Vector, CX3CL1-KD+ICAM-1, or control VBMECs (5 × 10^4^) to 100% confluence and formation of an endothelial monolayer barrier. Then, the tumor cells (2 × 10^4^) labeled with 2',7'-bis-(2-carboxyethyl)5-(and-6)-carboxyfluorescein acetoxymethyl ester (Fanbo) were seeded into the upper chamber, treated with or without the Src signaling pathway agonist tamoxifen (1 µM). After 24 h, cells that translocated to the lower membrane were observed and captured by a fluorescence microscope (Olympus BX53). Assays were quantified by counting the number of stained cells in five randomly selected fields in each well.

### Fluorescein isothiocyanate (FITC)-labeled transendothelial dextran flux

CX3CL1-KD, ICAM-1-KD, CX3CL1-KD+Vector, CX3CL1-KD+ICAM-1, or control VBMECs (5 × 10^4^) were seeded into the upper chambers of a Transwell system (cat. no. 3402; Corning, Inc.). After reaching 100% confluence, a monolayer endothelial cell barrier was formed. FITC-labeled dextran (MW 70,000; Seebio, Shanghai, China) was added to the upper chamber at a concentration of 100 µg/mL. Conditioned media from A549 cells, with or without tamoxifen (1 µM), was added to the lower and upper chambers and cells were incubated for 2 h. A total of 100 µL of medium was harvested from the lower and upper chambers following measurement of fluorescence intensity and calculation of the transendothelial dextran flux rate (%/h/cm^2^).

### Animal model

The protocol for the animal experiments was approved by the Animal Ethics Committee of Zhongshan Hospital, Fudan University. Eighty 4-6-week-old male NOD/SCID mice (14-20 g) were obtained from Vital River Laboratory Animal Technology Co., Ltd. (Beijing, China) and randomly assigned to eight groups. The mice were anesthetized by intraperitoneal injection of sodium pentobarbital (80 mg/kg). For metastasis studies, 1 × 10^6^ shCX3CR1-A549, 1 × 10^6^ shLFA-1-A549, 1 × 10^6^ shCX3CR1- and shLFA-1-A549, 1 × 10^6^ shCX3CR1+Vector-A549, 1 × 10^6^ shCX3CR1+LFA-1-A549, or 1 × 10^6^ control A549 cells were injected into the left cardiac ventricle of anesthetized mice. Development of metastases was monitored by bioluminescence (BLI) every week. BLI images were captured with a Xenogen IVIS 200 Imaging System. Analysis was conducted by measuring photon flux in the spines of mice using Living Image software and data were normalized to the signal. After 6‑8 weeks, the mice were sacrificed by intraperitoneal injection of sodium pentobarbital (200 mg/kg) and scanned by micro-computerized tomography (micro‑CT). Subsequently, spines were excised from mice and immersed in 4% paraformaldehyde for 7 days, decalcified in 10% EDTA for 2 weeks, embedded in paraffin, and dissected in the midsagittal direction. Then, 4 μm thick sections were prepared and subjected to hematoxylin and eosin (HE) staining and immunohistochemistry as previously described 13.

### Statistical analysis

Data are presented as the mean ± SEM or SD as indicated in figure legends. At least triplicate parallel samples were collected for each set of results. One-way analysis of variance (ANOVA) or two-tailed Student's *t*-test using SPSS 25.0 software (SPSS, Inc., Chicago, IL, USA) was used to analyze differences among groups. Comparisons between Kaplan-Meier curves were performed using the log-rank test. A *P*-value of less than 0.05 was considered statistically significant (*P < 0.05, **P < 0.01, and ***P < 0.001).

## Results

### CX3CL1 expression level is specifically higher in vertebral bone compared with limb bones and lung tissue

**Figure 1:**
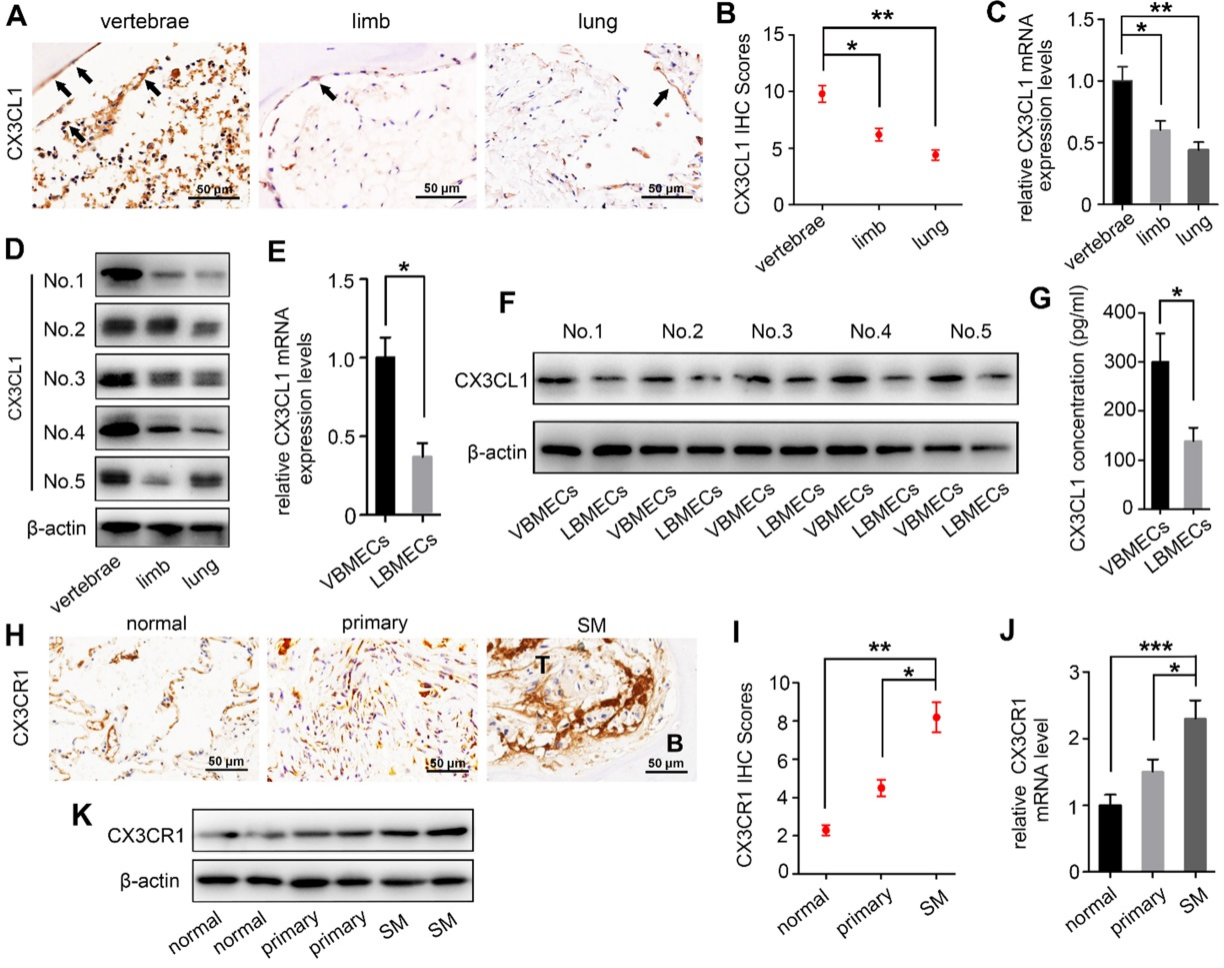
** CX3CL1 expression level is increased in vertebral bone and is associated with NSCLC spinal metastasis. (A, B)** Quantitative IHC analysis of CX3CL1 protein expression in cancellous bone of vertebrae (vertebrae, *n* = 5), cancellous bone of lower femur (limb, *n* = 5), and normal lung tissue (lung, *n* = 5). Representative micrographs of these samples are shown. Data represent the mean ± standard error of the mean (SEM). *P < 0.05 and **P < 0.01. **(C)** *CX3CL1* mRNA levels in cancellous bone of vertebrae, cancellous bone of the lower femur, and normal lung tissue. Data represent the mean ± SEM. *P < 0.05 and **P < 0.01.** (D)** Western blotting analysis of CX3CL1 protein levels in cancellous bone of vertebrae, cancellous bone of the lower femur, and normal lung tissue. Representative western blots of three independent experiments are shown.** (E)** RT-qPCR analysis of *CX3CL1* mRNA expression levels in bone marrow endothelial cells isolated from cancellous bone of vertebrae (VBMECs) and limb (LBMECs). Data represent the mean ± SEM. *P < 0.05.** (F)** CX3CL1 protein levels in VBMECs and LBMECs.** (G)** Concentration analysis of CX3CL1 in VBMEC- and LBMEC-cultured media indicated by ELISA. Data represent the mean ± SEM. *P < 0.05.** (H, I)** Quantitative IHC analysis of CX3CR1 protein expression in normal lung tissues (normal, *n* = 11), primary tumors (primary, *n* = 11), and spinal metastatic samples (SM, *n* = 11). Data represent the mean ± SEM. *P < 0.05 and **P < 0.01.** (J)** RT-qPCR of the *CX3CR1* mRNA expression levels in NSCLC spinal metastases, primary tumors and normal lung tissues. Data represent the mean ± SEM. *P < 0.05 and ***P < 0.001.** (K)** Western blot analysis of CX3CR1 levels in NSCLC spinal metastases, primary tumors and normal lung tissues.

There is increasing evidence that molecular signals in the tumor-associated bone marrow microenvironment facilitate cancer progression 18. We examined CX3CL1 expression in a spectrum of normal cancellous bone of vertebrae, cancellous bone of the lower femur, and normal lung tissue using immunohistochemistry and found dramatically elevated CX3CL1 expression in vertebral bone compared with visceral tissues (Figure 1A-B). Notably, the staining intensities in cancellous bone of vertebrae were also significantly greater than those in cancellous bone of the lower femur. RT-qPCR analysis consistently revealed remarkably higher mRNA expression levels of *CX3CL1* in vertebral bone than in visceral tissues and limbs (Figure 1C). Western blotting revealed that protein expression levels of CX3CL1 were highest in vertebral bone among tissues from different organs (Figure 1D). These findings demonstrated the local specificity of CX3CL1 distribution in vertebral bone.

Bone marrow endothelial cells (BMECs) are an important component of the bone marrow microenvironment and serve as a major source of cytokines in cancellous bone 14, 19, 20. To determine whether the specificity of CX3CL1/ICAM-1 distribution can be attributed to its discriminatory expression in VBMECs, mRNA and protein levels of CX3CL1 in LBMECs and VBMECs were detected. As shown in Figure 1E, the relative fold change of the *CX3CL1* mRNA level in VBMECs of passage three was significantly higher than that in LBMECs of passage three. Western blotting analysis revealed a remarkably higher CX3CL1 expression level in VBMECs compared with LBMECs (Figure 1F). Additionally, ELISA analysis showed that CX3CL1 protein secretion in VBMEC culture media was significantly higher than that in LBMEC culture, indicating alteration of the microenvironment in cancellous bone of vertebrae and the potential effects on NSCLC cell behavior (Figure 1G).

As a unique receptor of CX3CL1, CX3CR1 is a main effector of CX3CL1 in tumor cell behavior 21. To determine the relevance of higher CX3CL1 expression in vertebral cancellous bone in NSCLC spinal metastases, the expression of CX3CR1 in normal lung tissues, primary tumors, and spinal metastatic samples was detected. Immunohistochemistry demonstrated increased CX3CR1 protein expression in spinal metastases relative to primary tumors in nine of 11 cases (Figure 1H-I). The mRNA expression levels of *CX3CR1* were also higher in NSCLC spinal metastases than in primary tumors and normal lung tissues (Figure 1J). Western blot analysis further confirmed that CX3CR1 levels in NSCLC spinal metastases were higher than those in primary tumors and normal tissues (Figure 1K). These results collectively indicated that CX3CL1 was specifically distributed in vertebrae and that the higher CX3CL1 expression in VBMECs may play an essential role in facilitating metastasis of NSCLC to the spine via binding of CX3CR1 on tumor cells.

### VBMECs enhanced NSCLC cell invasion via CX3CL1 signaling-mediated activation of the PI3K/AKT pathway

**Figure 2:**
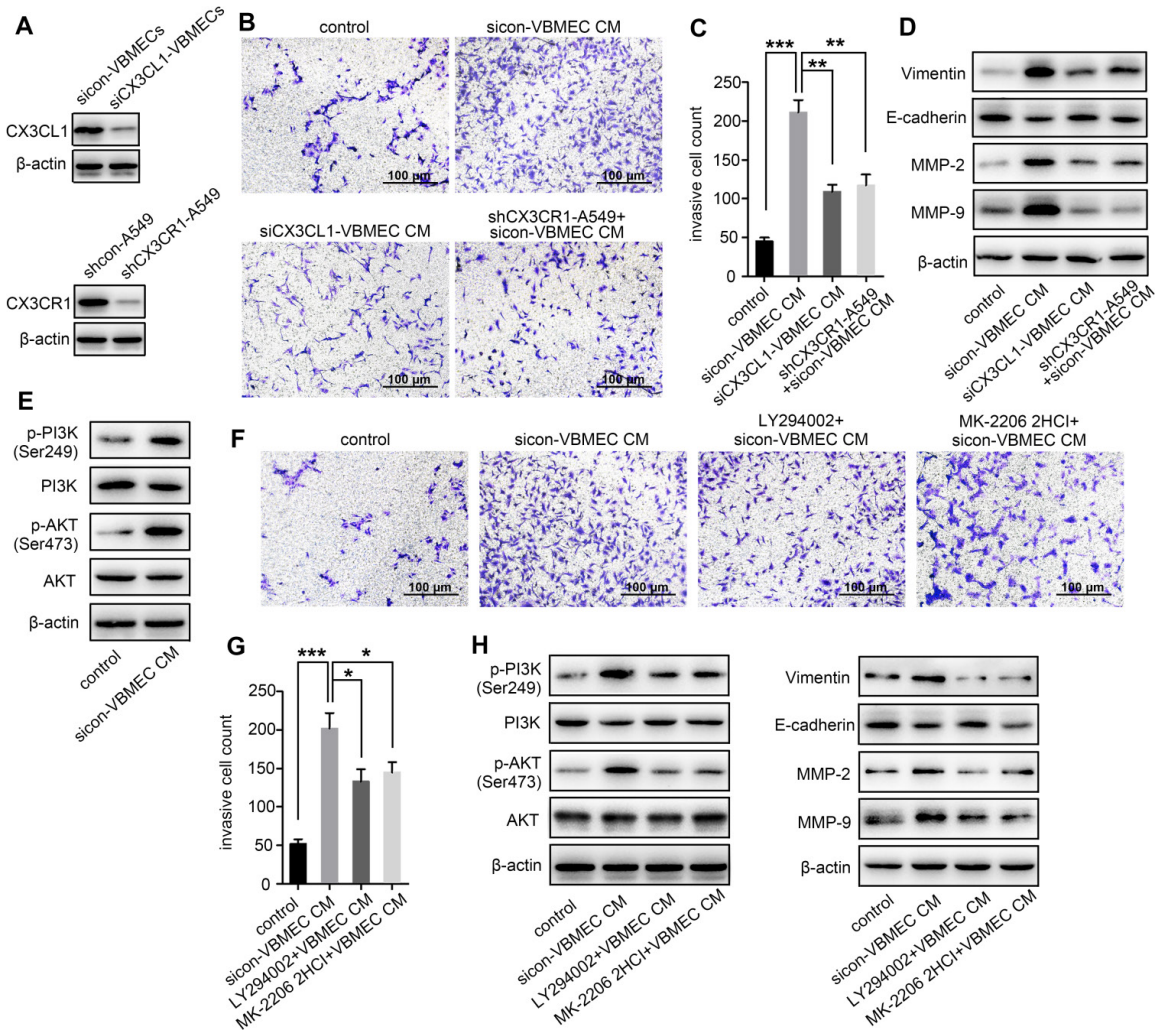
** VBMECs enhanced NSCLC cell invasion via CX3CL1 signaling-mediated activation of the PI3K/AKT pathway. (A)** Western blotting analysis of CX3CL1 protein levels in control (sicon) and CX3CL1-KD (siCX3CL1) VBMECs (Vs), and CX3CR1 protein levels in control (shcon) and CX3CR1-KD (shCX3CR1) A549 cells (A549). **(B, C)** Quantitative analysis of cell invasion in control (shcon) and CX3CR1-KD (shCX3CR1) A549 cells incubated with or without conditioned culture media of indicated VBMECs (VBMEC CM). Data represent the mean ± SEM (*n* = 3). **P < 0.01 and ***P < 0.001. **(D)** Expression levels of EMT markers including Vimentin, E-cadherin, MMP-2, and MMP-9 in control (shcon) and CX3CR1-KD (shCX3CR1) A549 cells co-cultured with or without indicated VBMEC conditioned media measured by western blotting. **(E)** PI3K and AKT phosphorylation in A549 cells co-cultured with or without VBMEC conditioned media measured by western blotting.** (F, G)** Quantitative analysis of invasion of A549 cells incubated with or without conditioned culture media of VBMECs treated with the PI3K inhibitor LY294002 or AKT inhibitor MK-2206 2HCI. Data represent the mean ± SEM (*n* = 3). *P < 0.05 and ***P < 0.001. **(H)** Expression levels of PI3K, p-PI3K (Ser249), AKT, p-AKT (Ser473), Vimentin, E-cadherin, MMP-2, and MMP-9 in A549 cells co-cultured with indicated VBMECs and treated with LY294002 or MK-2206 2HCI measured by western blotting.

As a chemokine, CX3CL1 has been shown to facilitate increased cancer cell invasion 14, 22. To investigate whether increased expression of CX3CL1 in VBMECs promotes NSCLC spinal metastasis by accelerating cell invasion, we incubated A549 cells with conditioned culture media of VBMECs to quantify NSCLC cell invasion using a Transwell assay. A549 cells grown in conditioned culture media showed an approximately 3-fold improvement in cell invasion compared with the control, paralleling a 3-fold increase in the aggressive nature of tumor cells relative to controls (Figure 2B-C). Genetic ablation of CX3CL1 in VBMECs minimized the increase in A549 cell invasion conferred by VBMECs (Figure 2A-C, *P* < 0.01). Furthermore, silencing CX3CR1 in A549 cells, which is the only receptor of CX3CL1, significantly reduced VBMEC-induced cell invasion (*P* < 0.01), indicating CX3CL1/CX3CR1 functionally mediated VBMEC's effect on NSCLC cell invasion. We were able to recapitulate and reverse VBMEC promotion of NCSLC invasion in another NSCLC cell line, H1975 (Figure S2A-B). These results suggested that VBMECs promoted NSCLC cell invasion via CX3CL1-dependent signaling pathways.

Tumor cell invasion is often associated with the epithelial-to-mesenchymal transition (EMT) 23. To investigate whether CX3CL1-mediated promotion of NSCLC cell invasion occurs via the EMT, we examined the expression levels of EMT markers in A549 cells following incubation in conditioned culture media of VBMECs. As shown in Figure 2D, co-culture with VBMECs significantly increased expression of the mesenchymal marker vimentin and decreased expression of the epithelial marker E-cadherin in A549 cells compared with controls. Further genetic ablation of CX3CL1 in VBMECs or of CX3CR1 in A549 cells evidently reduced vimentin expression and upregulated E-cadherin expression in A549 cells, reversing changes caused by VBMECs. We also analyzed the expression of several matrix metalloproteinases (MMPs), which are known to participate in degradation of the matrix, an essential part of cell invasion. As indicated in Figure 2D, MMP-2 and MMP-9 levels in A549 cells were significantly upregulated at 24 h post-treatment with conditioned media of VBMECs. Further genetic inhibition of CX3CL1 in VBMECs or of CX3CR1 in A549 cells consistently reduced the expression of MMP-2 and MMP-9 in NSCLC cells, abolishing the alterations mediated by VBMECs. These findings suggested that VBMECs upregulated NSCLC cell invasion through CX3CL1-dependent promotion of the EMT and MMP-mediated matrix degradation. Another human NSCLC cell line tested, H1975, had similar results (Figure S2C).

Activation of PI3K/AKT has been reported to regulate tumor cell migration and invasion through distinct ways, such as upregulating several transcription factors and enhancing MMP-mediated matrix degradation 24. To determine whether co-culture of VBMECs affects PI3K/AKT activation to improve NSCLC invasion, PI3K and AKT expression levels were detected. Co-culture with conditioned media of VBMECs effectively promoted PI3K and AKT phosphorylation in A549 cells without obvious changes in the total PI3K and AKT expression levels, indicating the involvement of PI3K and AKT phosphorylation in VBMEC-mediated promotion of the invasive capability of NSCLC cells (Figure 2E). To confirm the role of the PI3K/AKT pathway in NSCLC cell invasion regulated by VBMECs, the PI3K inhibitor LY294002 and AKT inhibitor MK-2206 2HCI were applied. Interestingly, LY294002 and MK-2206 2HCI treatment of conditioned media-treated A549 cells significantly inhibited the invasive ability compared with the conditioned media-treated controls, reversing changes caused by VBMECs (Figure 2F-G). Western blot analysis showed that decreased E-cadherin expression in conditioned media-treated A549 cells was recovered by LY294002 or MK-2206 2HCI, whereas upregulated Vimentin, MMP-2, and MMP-9 proteins were reduced by LY294002 or MK-2206 2HCI treatments (Figure 2H). Related assays with another NSCLC cell line, H1975, showed similar results (Figure S2D-H). Overall, these data demonstrated that VBMECs conferred an NSCLC cell invasion advantage via activation of the PI3K/AKT pathway in a CX3CL1/CX3CR1-dependent manner.

### VBMECs induced ICAM-1-dependent NSCLC cell adhesion in coordination with platelets through the CX3CL1/ICAM-1/LFA-1 pathway

**Figure 3:**
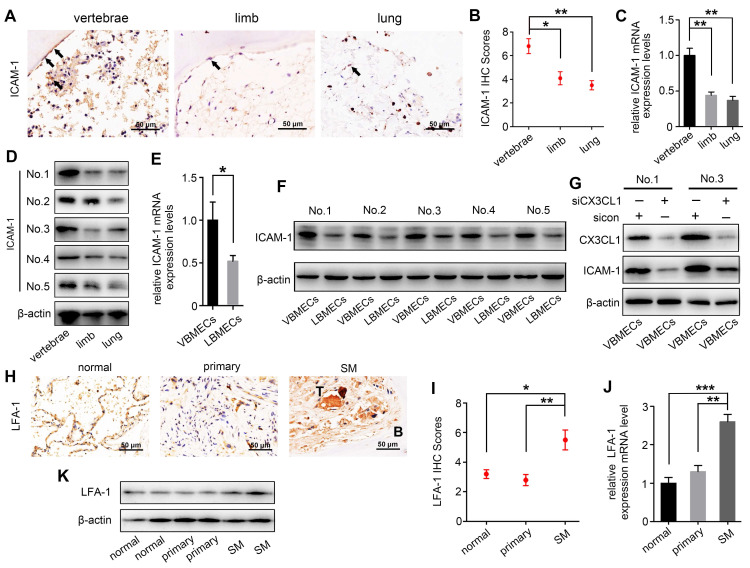
** ICAM-1/LFA-1 expression is associated with CX3CL1-mediated NSCLC spinal metastases. (A, B)** Quantitative IHC analysis of ICAM-1 protein expression in cancellous bone of vertebrae (vertebrae, *n* = 5), cancellous bone of lower femur (limb, *n* = 5), and normal lung tissue (lung, *n* = 5). Representative micrographs of these samples are shown. Data represent the mean ± SEM. *P < 0.05 and **P < 0.01. **(C)** *ICAM-1* mRNA levels in vertebrae, limb, and lung tissues. Data represent the mean ± SEM. **P < 0.01.** (D)** Western blotting analysis of ICAM-1 protein levels in vertebrae, limb, and lung tissues. **(E)** RT-qPCR of the *ICAM-1* mRNA expression levels in VBMECs and LBMECs. Data represent the mean ± SEM. *P < 0.05.** (F)** CX3CL1 protein levels in VBMECs and LBMECs detected by western blotting.** (G)** Genetic ablation of CX3CL1 (siCX3CL1) significantly decreased ICAM-1 expression in VBMECs indicated by western blotting.** (H, I)** Quantitative IHC analysis of LFA-1 protein expression in normal lung tissues (normal, *n* = 11), primary tumors (primary, *n* = 11), and spinal metastatic samples (SM, *n* = 11). Data represent the mean ± SEM. *P < 0.05 and **P < 0.01.** (J)** RT-qPCR of the *LFA-1* mRNA expression levels in NSCLC spinal metastases, primary tumors and normal lung tissues. Data represent the mean ± SEM. **P < 0.01 and ***P < 0.001.** (K)** Western blot analysis of LFA-1 levels in NSCLC spinal metastases, primary tumors and normal lung tissues.

ICAM-1 is an important adhesion molecule, and data have shown that ICAM-1 is involved in cancer progression and promotes CX3CL1-mediated metastasis of various tumors 25, 26. To determine whether ICAM-1 was involved in CX3CL1-induced NSCLC spinal metastasis, ICAM-1 expression in vertebral bone and NSCLC spinal metastases was examined. Immunohistochemistry showed that the ICAM-1 protein level in the cancellous bone of vertebrae was significantly higher than that in the cancellous bone of limbs and visceral tissues (Figure 3A-B). RT-qPCR analysis revealed higher mRNA levels of *ICAM-1* in vertebral bone than in limb and lung (Figure 3C). Western blotting analysis further confirmed the higher expression of ICAM-1 in vertebral bone (Figure 3D). ICAM-1 is thought to be mainly expressed on endothelial cells; therefore, ICAM-1 protein levels in VBMECs and LBMECs were further detected. As indicated in Figure 3E and 3F, western blotting and RT-qPCR analyses consistently showed higher expression levels of ICAM-1 in VBMECs compared with LBMECs, suggesting that the discriminatory distribution of ICAM-1 was attributed to its different expression levels in BMECs. Furthermore, genetic inhibition of CX3CL1 in VBMECs significantly reduced the expression of ICAM-1, indicating ICAM-1 was a downstream effector of CX3CL1 signaling in VBMECs (Figure 3G). It has been reported that the main ligand of ICAM-1 on target tumor cells is LFA-1; thus, expression of LFA-1 in the clinical samples was further investigated 25, 27. As shown in Figure 3H and 3I, LFA-1 was highly expressed in NSCLC spinal metastases compared with primary tumors and normal lung tissues, as shown by the immunohistochemistry results. RT-qPCR analysis also revealed higher expression of* LFA-1* in spine-metastatic lesions than in primary tumors and healthy lung tissues (Figure 3J). Western blotting analysis further confirmed this phenomenon (Figure 3K). Collectively, these findings suggested that ICAM-1/LFA-1 may play an important role in CX3CL1-mediated NSCLC spinal metastasis.

**Figure 4:**
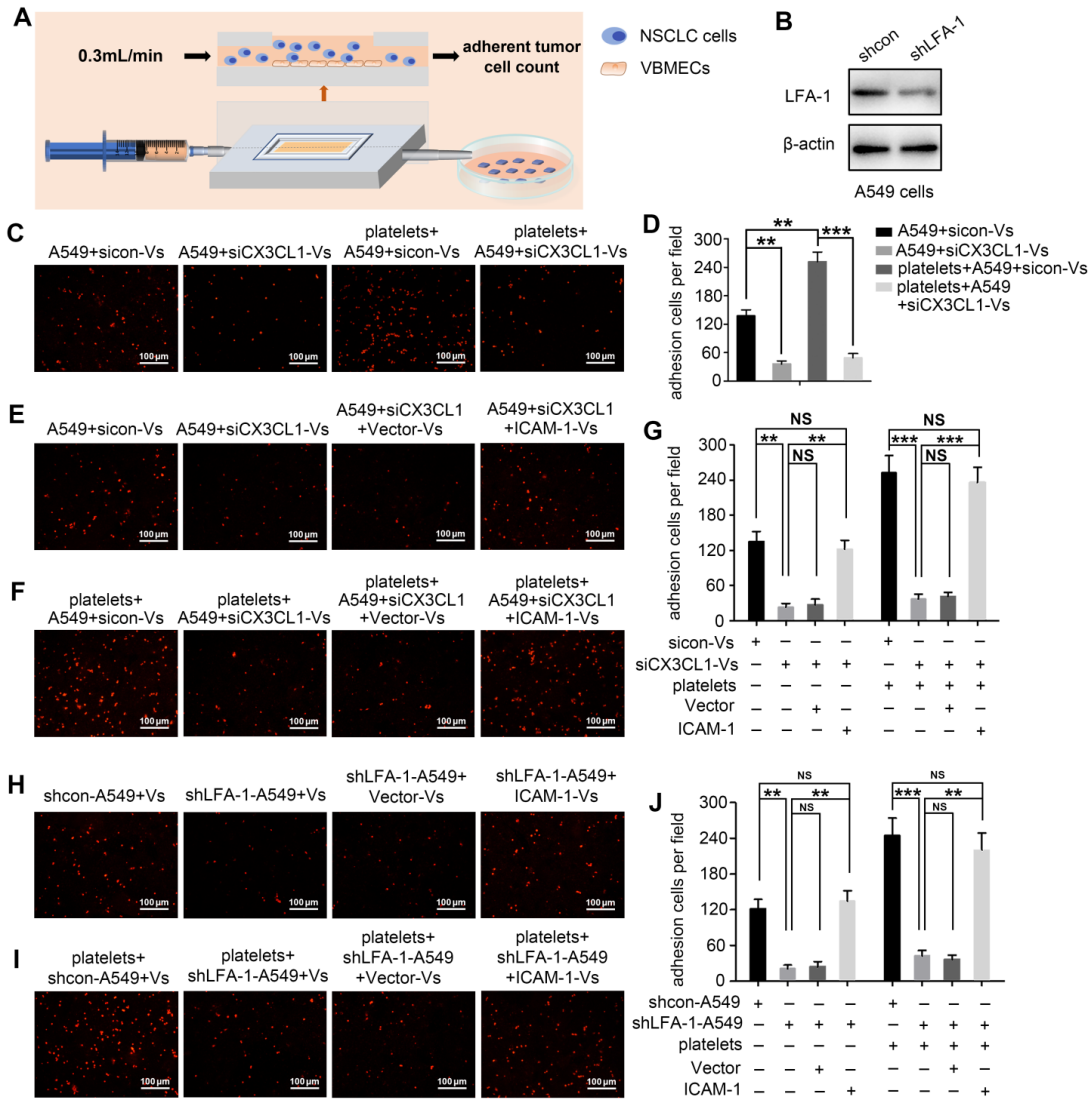
** VBMECs induced NSCLC cell adhesion through the CX3CL1/ICAM-1/LFA-1 pathway in the presence or absence of platelets. (A)** Schematic illustration of the parallel-plate flow chamber system for adhesion assays. **(B)** Genetic inhibition of LFA-1 in A549 cells (shLFA-1). **(C, D)** Quantitative analysis of NSCLC cell adhesion to indicated VBMECs in the presence or absence of platelets. Data represent the mean ± SEM (*n* = 3). **P < 0.01, and ***P < 0.001.** (E-G)** Overexpression of ICAM-1 in VBMECs remarkably rescued the efficacy of CX3CL1 inhibition to decrease NSCLC cell adhesion to VBMECs. Data represent the mean ± SEM (*n* = 3). **P < 0.01, ***P < 0.001, and^ NS^*P* > 0.05.** (H-J)** Overexpression of ICAM-1 in VBMECs significantly reversed the decrease in NSCLC cell adhesion to VBMECs induced by LFA-1-KD treatment. Data represent the mean ± SEM (*n* = 3). **P < 0.01, ***P < 0.001, and ^NS^*P* > 0.05.

Considering that CX3CL1 is an important chemokine and ICAM-1 is an important adhesion molecule, we hypothesized that VBMECs recruit circulating NSCLC cells via CX3CL1/ICAM-1-induced cell adhesion. To investigate the effect of CX3CL1/ICAM-1 on NSCLC cell adhesion, a parallel-plate flow chamber system with monolayer CX3CL1-KD or control VBMECs serving as the fixed phase was applied (Figure 4A). As shown in Figure 4C and 4D, there was a large number of adhered cells on the stationary phase in the sicon-VBMEC group (138 ± 13 cells/well) while genetic inhibition of CX3CL1 in VBMECs obviously decreased the number of adhered cells on the fixed phase (41 ± 7 cells/well, *P* < 0.01), indicating the role of CX3CL1 in mediating circulating NSCLC cell adhesion to VBMECs. Notably, addition of platelets to the system elicited a robust increase in the number of adhered cells in control VBMEC groups, which was abolished by genetic silencing of CX3CL1 in VBMECs, suggesting that platelets provided NSCLC cell adhesion advantages in a CX3CL1-dependent manner. Another human NSCLC cell line tested, H1975, had similar results (Figure S3A-B). However, following administration of ICAM-1 overexpression, the number of adhered cells remarkably increased in the CX3CL1-KD group, and there was no significant discrepancy between the ICAM-1-overexpression and control VBMEC groups, indicating ICAM-1 as a main downstream effector in CX3CL1-induced NSCLC cell adhesion (Figure 4E and 4G). Meanwhile, overexpression of ICAM-1 also significantly increased the number of adhered tumor cells and reversed the adhesion disadvantages of NSCLC cells conferred by CX3CL1-KD treatment in the presence of platelets, highlighting the dependent role of ICAM-1 in platelet-assisted NSCLC cell adhesion (Figure 4F-G). Another NSCLC cell line with ICAM-1 overexpression, H1975, showed similar results (Figures S3C-E).

LFA-1 is a primary receptor of ICAM-1. To determine whether CX3CL1-mediated circulating NSCLC cell adhesion to VBMECs occurred via ICAM-1 binding to LFA-1, the LFA-1 gene in A549 and H1975 cells was knocked-down using LFA-1 shRNA while the ICAM-1 gene in VBMECs was overexpressed using ICAM-1-overexpressing lentiviruses (Figure 4B, Figure S1B and S1D). As shown in Figure 4H and Figure S3F, genetic ablation of LFA-1 significantly reduced the adhesion of NSCLC cells to VBMECs. In the presence of platelets, genetic inhibition of LFA-1 also remarkably decreased the adhesion of NSCLC cells to VBMECs (Figure 4I and Figure S3G). Interestingly, when the ICAM-1 gene was overexpressed in VBMECs, the adhesion disadvantages of NSCLC cells conferred by LFA-1 silencing were evidently attenuated, rescuing the ability of LFA-1 KD to decrease NSCLC cell adhesion to VBMECs (Figure 4H-J and Figure S3F-H). These data indicated that LFA-1 was a crucial downstream effector of ICAM-1-mediated NSCLC cell adhesion in the presence or absence of platelets. Taken together, these results suggested that VBEMCs functionally induced circulating NSCLC cell adhesion to the vertebral microvascular endothelium in coordination with platelets via the CX3CL1/ICAM-1/LFA-1 pathway.

### CX3CL1 enhanced NSCLC cell transendothelial migration by increasing permeability of VBMECs via ICAM-1-dependent activation of the Src/GEF-H1 pathway

**Figure 5:**
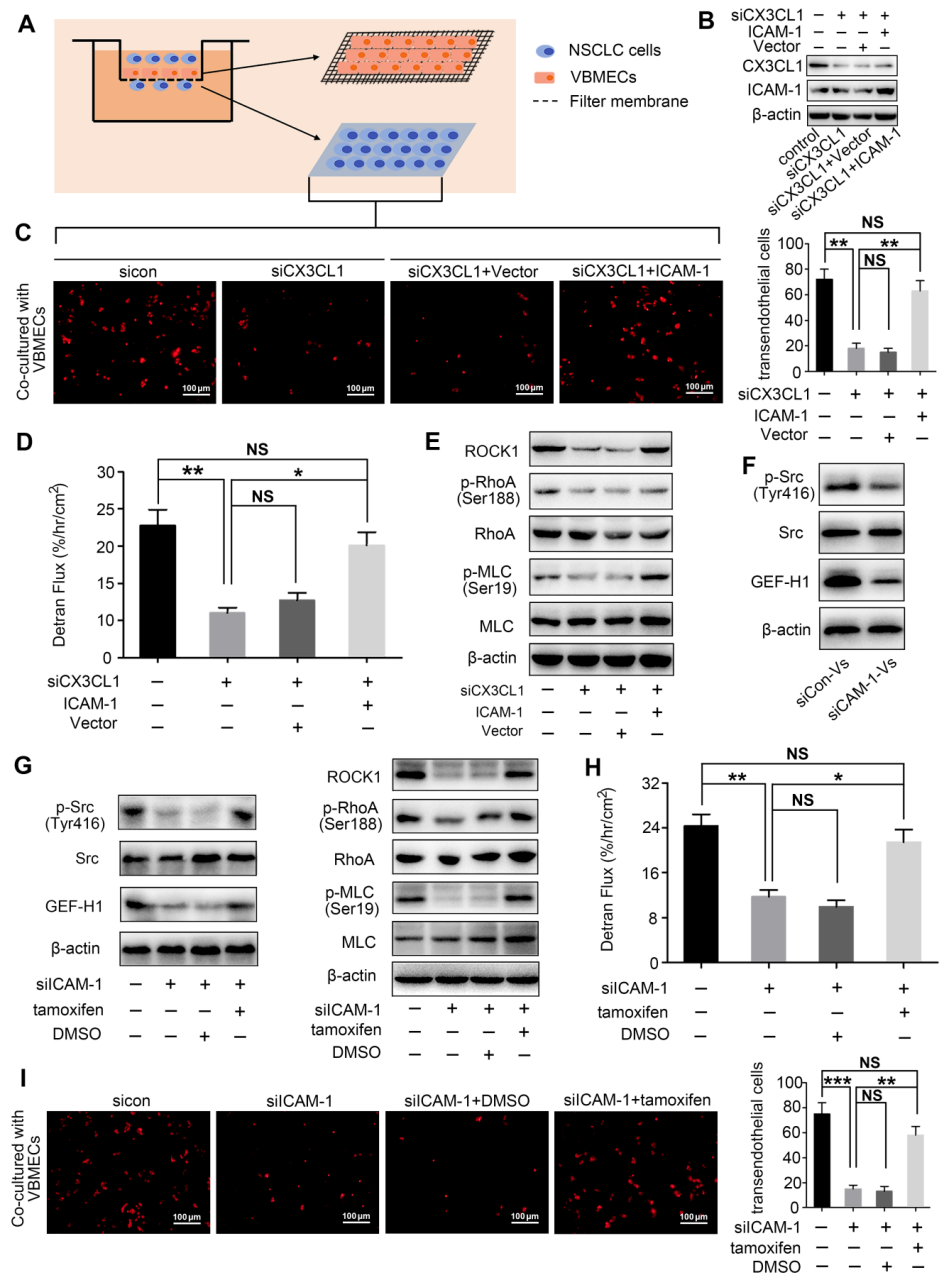
** CX3CL1/ICAM-1 enhanced NSCLC cell transendothelial migration by increasing VBMEC permeability through the Src/GEF-H1 pathway. (A)** Schematic illustration of the Transwell analysis of transendothelial migration assays.** (B)** Western blotting analysis of CX3CL1 and ICAM-1 protein levels in indicated VBMECs.** (C)** Quantitative analysis of A549 cell transendothelial migration with control (sicon), CX3CL1-KD (siCX3CL1), CX3CL1-KD and Vector (siCX3CL1+Vector) or CX3CL1-KD and ICAM-1-overexpression (siCX3CL1+ICAM-1) VBMECs as an endothelial monolayer barrier. Data represent the mean ± SEM (*n* = 3). **P < 0.01 and ^NS^*P* > 0.05. **(D)** Transport of FITC-labeled dextran through the indicated VBMEC barrier treated with conditioned media from A549 cells. Data represent the mean ± SEM (*n* = 3). *P < 0.05, **P < 0.01, and ^NS^*P* > 0.05. **(E)** Western blotting of RhoA, p-RhoA, MLC, p-MLC, and ROCK1 protein levels in the indicated VBMECs treated with conditioned media from A549 cells. **(F)** Src, p-Src, and GEF-H1 levels in the indicated VBMECs treated with conditioned media from A549 cells detected by western blotting analysis. **(G)** Western blotting of RhoA, p-RhoA, MLC, p-MLC, and ROCK1 protein levels in the indicated VBMECs incubated with conditioned media from A549 cells, treated with the Src signaling pathway agonist tamoxifen. **(H)** Transport of FITC-labeled dextran through the indicated VBMEC barrier induced by conditioned media from A549 cells and treated with tamoxifen. Data represent the mean ± SEM (*n* = 3). *P < 0.05, **P < 0.01, and ^NS^*P* > 0.05. **(I)** Quantitative analysis of NSCLC cell transendothelial migration with administration of tamoxifen in the indicated co-cultures of VBMECs and A549 cells. Data represent the mean ± SEM (*n* = 3). **P < 0.01, ***P < 0.01, and ^NS^*P* > 0.05.

Transendothelial migration is a crucial step in tumor metastasis; thus, the effect of CX3CL1 on NSCLC cell transendothelial migration was investigated (Figure 5A). As indicated in Figure 5C and Figure S4B, the number of NSCLC cells crossing the CX3CL1-KD VBMEC barrier was significantly lower than the number of cells crossing the control VBMEC barrier, necessitating CX3CL1 for NSCLC cell transendothelial migration. Furthermore, ICAM-1-overexpression treatment in VBMECs rescued the efficacy of CX3CL1-KD inhibition of NSCLC cell transendothelial migration, indicating ICAM-1 signaling was required for CX3CL1-mediated NSCLC cell transendothelial migration (Figure 5B-C, and Figure S4A-B). To investigate the mechanisms involved in CX3CL1/ICAM-1-mediated NSCLC cell transendothelial migration, FITC-labeled transendothelial dextran flux for detecting permeability of monolayer endothelial cells was used. As shown in Figure 5D, genetic ablation of CX3CL1 significantly suppressed dextran transport, while overexpression of ICAM-1 remarkably rescued the efficacy of CX3CL1 ablation in decreasing transendothelial dextran transport. Western blot analysis indicated that genetic inhibition of CX3CL1 significantly reduced the expression of Rho-associated kinase 1 (ROCK1) and decreased the phosphorylation of Ras Homolog Family Member A (RhoA) and myosin light chain (MLC) in VBMECs, which was reversed by ICAM-1-overexpression (Figure 5E and Figure S4C). Considering that transendothelial dextran transport is a direct reflection of VBMEC permeability, and that ROCK1, RhoA, and MLC are key molecules in regulation of the cell cytoskeleton, we suggested that CX3CL1 can effectively upregulate VBMEC permeability in an ICAM-1-dependent manner, which led to the increased transendothelial migration of NSCLC cells.

Given that CX3CL1 and ICAM-1 do not directly regulate the permeability of VBMECs, there should be other signaling pathways responsible for alteration of VBMEC permeability. Evidence indicated that the binding of ICAM-1 to its ligands in endothelial cells induced an increase in intracellular Ca^2+^ concentration, thus promoting activation of Rho protein, which is associated with cytoskeleton organization and endothelium permeability 28, 29. Additionally, previous studies showed that the increase in ICAM-1 expression, accompanied by an increase in Src phosphorylation, promoted the expression of Rho guanine nucleotide exchange factor (GEF)-H1 30, 31, which has been demonstrated to improve endothelium permeability by regulating the kinase activity of RhoA 32. Therefore, activation of the Src/GEF-H1 signaling pathway in CX3CL1/ICAM-1-mediated regulation of VBMEC permeability was detected. As shown in Figure S1C, Figure 5F-G and Figure S4D-F, genetic ablation of ICAM-1 significantly reduced the phosphorylation of Src and expression of GEF-H1, as well as decreased the levels of ROCK1, p-RhoA, and p-MLC. Moreover, when the Src signaling pathway agonist tamoxifen was applied, the reductions in the levels of ROCK1, p-RhoA, and p-MLC induced by ICAM-1 silencing were significantly reversed (Figure 5G and Figure S4F). Additionally, dextran transport was inhibited by ICAM-1-KD treatment; this effect was reversed by treatment with tamoxifen (Figure 5H). Furthermore, transendothelial migration of tumor cells was remarkably decreased by inhibition of ICAM-1; this was also reversed by administration of tamoxifen (Figure 5I and Figure S4G). Collectively, we concluded that CX3CL1 could induce NSCLC cell transendothelial migration by increasing the permeability of VBMECs via ICAM-1-dependent activation of the Src/GEF-H1 pathway.

### NSCLC cells promoted CX3CL1 secretion of VBMECs via MAPK14/ADMA17-dependent CX3CL1 release and NF-κB-dependent CX3CL1 synthesis

**Figure 6:**
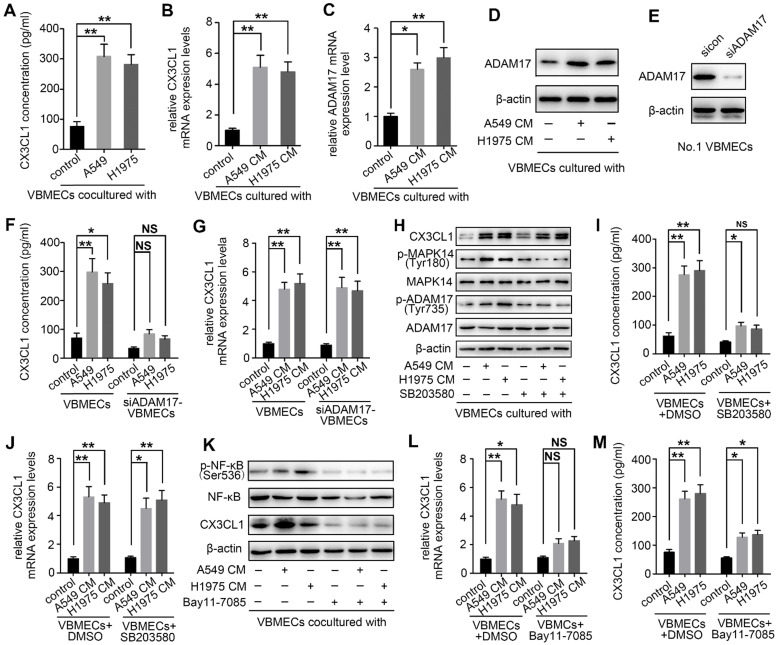
** NSCLC cells promoted CX3CL1 secretion of VBMECs by promoting MAPK14/ADMA17-dependent protein release and enhancing NF-κB-dependent CX3CL1 synthesis. (A)** Quantification of CX3CL1 concentration in culture media of the indicated co-cultures composed of VBMECs and H1975 or A549 cells by ELISA. Data represent the mean ± SEM (*n* = 3). **P < 0.01. **(B)** RT-qPCR analysis of *CX3CL1* mRNA levels in VBMECs co-cultured with culture media of NSCLC cell lines, A549 and H1975. Data represent the mean ± SEM (*n* = 3). **P < 0.01.** (C)** *ADMA17* mRNA levels in VBMECs co-cultured with culture media of NSCLC cell lines by RT-qPCR. Data represent the mean ± SEM (*n* = 3). *P < 0.05 and **P < 0.01.** (D)** Western blotting analysis of ADMA17 protein levels in VBMECs co-cultured with the indicated culture media of NSCLC cell lines.** (E)** Genetic silencing of ADAM17 in VBMECs.** (F)** Quantification of CX3CL1 secretion in culture media of indicated co-cultures combining ADAM17-KD VBMECs and H1975 or A549 cells by ELISA. Data represent the mean ± SEM (*n* = 3). *P < 0.05, **P < 0.01, and ^NS^*P* > 0.05.** (G)** RT-qPCR analysis of *CX3CL1* in the indicated VBMECs co-cultured with the indicated culture media of NSCLC cell lines. Data represent the mean ± SEM (*n* = 3). **P < 0.01.** (H)** Western blotting analysis of CX3CL1, MAPK14, p-MAPK14, ADAM17, and p-ADAM17 protein levels in VBMECs treated with conditioned media from A549 or H1975 cells in the presence or absence of the MAPK14 inhibitor SB203580. **(I)** Quantification of CX3CL1 concentration in culture media of the indicated co-cultures composed of VBMECs and H1975 or A549 cells treated with SB203580 by ELISA. Data represent the mean ± SEM (*n* = 3). *P < 0.05, **P < 0.01, and ^NS^*P* > 0.05. **(J)** RT-qPCR analysis of *CX3CL1* mRNA levels in VBMECs co-cultured with culture media of NSCLC cell lines and treated with SB203580**. (K)** Western blotting analysis of NF-κB, p-NF-κB, and CX3CL1 protein levels in VBMECs treated with conditioned media from A549 or H1975 cells in the presence or absence of the NF-κB inhibitor Bay11-7085. **(L)** RT-qPCR analysis of *CX3CL1* in the indicated VBMECs co-cultured with the indicated culture media of NSCLC cell lines and treated with Bay11-7085. Data represent the mean ± SEM (*n* = 3). *P < 0.05, **P < 0.01, and ^NS^*P* > 0.05.** (M)** Quantification of CX3CL1 concentration in culture media of the indicated co-cultures composed of VBMECs and H1975 or A549 cells treated with Bay11-7085 by ELISA. Data represent the mean ± SEM (*n* = 3). *P < 0.05 and **P < 0.01.

Upon arriving at the metastatic site, tumor cells can influence gene expression of endothelial cells, thereby inducing tumor metastasis 33. To determine whether NSCLC cells interact with VBMECs via modulation of CX3CL1 production, two kinds of NSCLC cell lines were co-cultured with VBMECs for 6 days followed by ELISA. As shown in Figure 6A, CX3CL1 concentrations in co-cultured media were 308.42 ± 40.35 pg/mL in the A549 group and 282.66 ± 31.59 pg/mL in the H1975 group, which were both nearly 4-fold those in single VBMEC cultures (76.33 ± 15.02 pg/mL, *P* < 0.01, *P* < 0.01), indicating the enhanced effect of tumor cells on CX3CL1 production in VBMECs. *CX3CL1* mRNA levels in VBMECs were also upregulated when co-cultured with NSCLC cell conditioned media (Figure 6B). However, the underlying mechanisms are poorly understood. It was reported that CX3CL1 possesses a mucin‑like domain that contains a cleavage site that allows metalloproteases, such as ADAM17, to cleave and release a soluble form of CX3CL1 34. Additionally, ADAM17 has been implicated in bone metastasis and associated with poor clinical outcomes 14. Therefore, the expression of ADAM17 in VBMECs co-cultured with or without NSCLC cells was detected. As seen in Figure 6C, *ADAM17* mRNA levels showed 2.6-fold and 3.0-fold increases in VBMECs cultured with conditioned media of A549 and H1975 cells compared with control VBMECs, respectively. Western blotting analysis revealed that culture media of both A549 and H1975 cells promoted the protein levels of ADAM17 in VBMECs (Figure 6D). Finally, we tested whether increased expression of ADAM17 in VBMECs accounted for increased CX3CL1 concentration in co-culture. As indicated in Figure 6E and 6F, ablation of ADAM17 in VBMECs diminished the increase in CX3CL1 concentration induced by co‑culturing VBMECs with A549 or H1975 cells. Notably, RT-qPCR analysis revealed that ablation of ADAM17 did not significantly reverse the improvement of *CX3CL1* expression in VBMECs co-cultured with NSCLC cells (Figure 6G). These results suggested that NSCLC cells stimulated secretion of soluble CX3CL1 via ADAM17-mediated CX3CL1 shedding from VBMECs.

Previous studies have reported that activation of ADAM17 depends on phosphokinases, such as MAPK14 14, 35. Therefore, the role of MAPK14 in ADAM17‑mediated production of CX3CL1 was further investigated. As shown in Figure 6H, co‑culturing VBMECs with A549 or H1975 cells significantly promoted the phosphorylation of MAPK14 (Tyr180) and ADAM17 (Tyr735). Treatment with the MAPK14 inhibitor SB203580 (0.5 µM) in the co-culture system effectively inhibited the activation of ADAM17 and minimized the increase in CX3CL1 concentration in co-cultured media conferred by NSCLC cells, indicating phosphorylated MAPK14 is a master effector in the activation of ADAM17 (Figure 6H-I).

Though inhibition of the MAPK14/ADAM17 pathway significantly attenuated the CX3CL1 secretion advantage of VBMECs conferred by tumor cells, CX3CL1 expression in VBMECs was still upregulated in the co-culture system compared with controls, even in the presence of the MAPK14 inhibitor SB203580 or with ADAM17 inhibition (Figure 6G-H, and 6J). These findings indicated the potential involvement of other tumor cell-responsive gene(s) in regulating CX3CL1 production. Nuclear factor kappa B (NF-κB) has been implicated in CX3CL1 signaling and to promote progression of several kinds of tumors 36, 37. As indicated in Figure 6K, the phosphorylation of NF-κB in VBMECs co-cultured with A549 or H1975 was significantly upregulated compared with control VBMECs. Treatment with the NF-κB inhibitor Bay11-7085 (10 µM) significantly minimized tumor cell-induced elevations of CX3CL1 expression. RT-qPCR analysis further confirmed the changes in *CX3CL1* expression following treatment with Bay11-7085 (Figure 6L). Meanwhile, ELISA results indicated that increased CX3CL1 concentration in co-culture media of VBMECs and NSCLC cells was reversed by Bay11-7085 treatment (Figure 6M). Collectively, these results demonstrated that NSCLC cells enhanced CX3CL1 expression in VBMECs by stimulating activation of NF-κB. Considering that NSCLC cells promoted CX3CL1 shedding from VBMECs by activating MAPK14/ADMA17 signaling, we suggested that NSCLC cells stimulated CX3CL1 secretion from VBMECs not only by enhancing MAPK14/ADMA17-mediated protein release but also by promoting NF-κB-dependent CX3CL1 synthesis.

### Disengagement of the CX3CL1/ICAM-1-mediated feedback cycle between circulating NSCLC cells and VBMECs attenuated NSCLC spinal metastasis* in vivo.*

**Figure 7:**
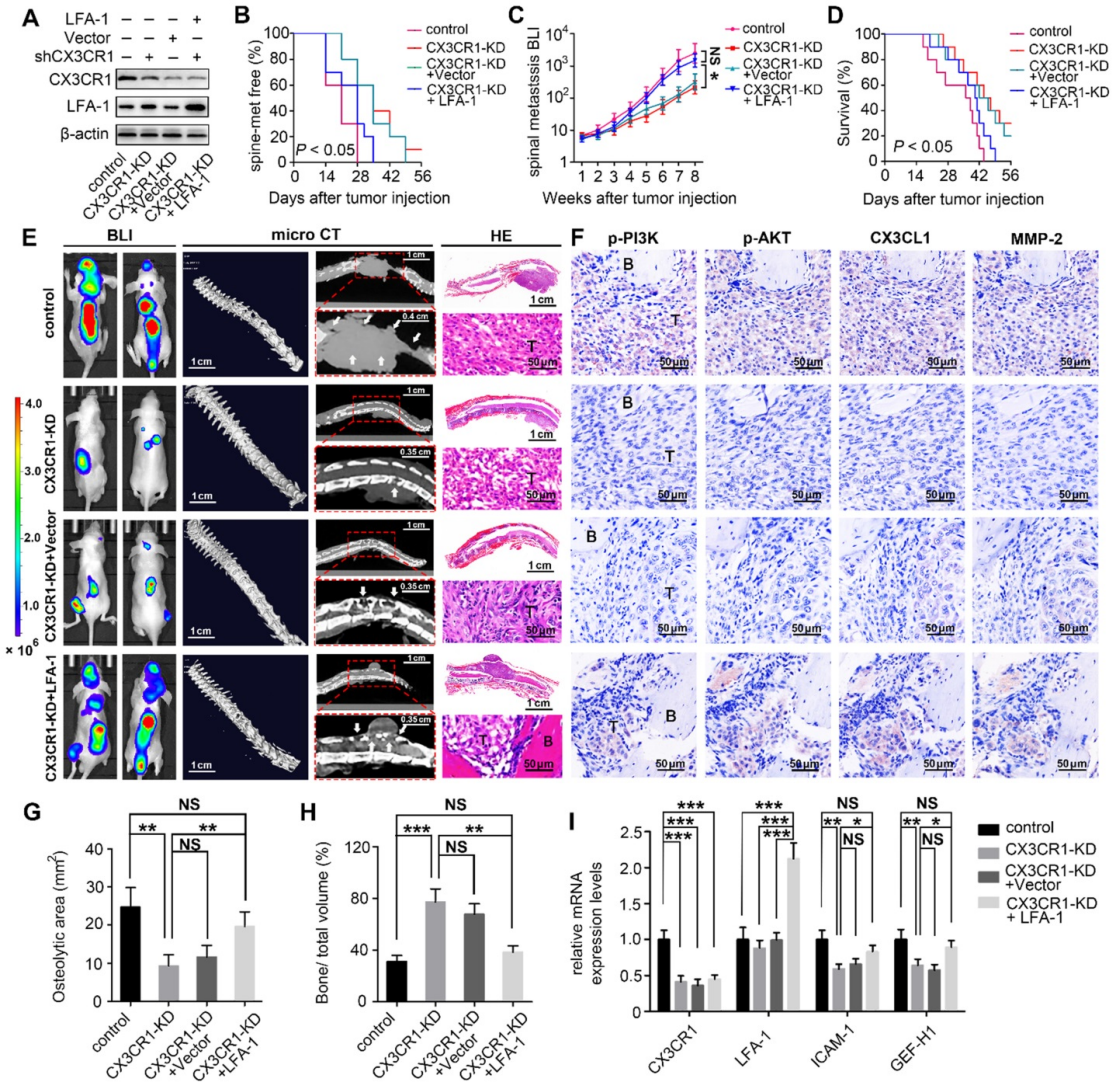
** Inhibition of the CX3CL1/ICAM-1-mediated feedback cycle between circulating NSCLC cells and VBMECs by silencing CX3CR1 in A549 cells reduced NSCLC spinal metastasis and prolonged survival in mice. (A)** Western blotting of CX3CR1 and LFA-1 protein levels in the indicated A549 cells. **(B)** Kaplan-Meier spinal metastasis-free curve of mice inoculated intracardially with control, CX3CR1-KD, CX3CR1-KD and Vector (CX3CR1-KD+Vector) or CX3CR1-KD and LFA-1-overexpression (CX3CR1-KD+LFA-1) A549 cells (*n* = 10). **(C)** Normalized BLI signals of spinal metastases. Data represent the mean ± standard deviation (SD) (*n* = 10). *P < 0.05 and ^NS^*P* > 0.05.** (D)** Kaplan-Meier survival curve of mice. **(E)** Representative BLI, microCT, and histological (HE) images of bone lesions (B) invaded by tumors (T) in each group. Arrows indicate osteolytic bone lesions and dashed rectangles are used to mark the regions of interest for vertebral bone scan and analysis. **(F)** IHC images of p-PI3K, p-AKT, CX3CL1, and MMP-2 in tumor areas from serial sections of the indicated samples.** (G, H)** Quantification of osteolytic areas of spine and bone volume relative to total volume from microCT scans. Data represent the mean ± SEM (*n* = 5). **P < 0.01, ***P < 0.001, and ^NS^*P* > 0.05. **(I)** mRNA levels of *CX3CR1*, *LFA-1*, *Icam-1*, and *Gef-h1* in tumors of mice by RT-qPCR. Data represent the mean ± SEM (*n* = 10). *P < 0.05, **P < 0.01, ***P < 0.001, and ^NS^*P* > 0.05.

The* in vitro* results revealed a feedforward loop whereby CX3CL1/ICAM-1 signaling in VBMECs stimulated adhesion of NSCLC cells to the microvascular endothelium while the increasing NSCLC cells in turn enhanced the tumor cell-induced CX3CL1 secretion of VBMECs, during which, the invasion of tumor cells was upregulated and the permeability of VBMECs was increased. The feedforward loop functionally facilitates NSCLC cell dynamic transmigration to cancellous bone of vertebrae. These data highlighted the pivotal role of CX3CL1/ICAM-1 in the interactions between tumor cells and VBMECs to create a vicious feedback cycle for NSCLC spinal metastasis. Given that CX3CR1 and LFA-1 functionally mediated the effect of CX3CL1/ICAM-1 on NSCLC cell behavior, nude mice were inoculated intracardially with CX3CR1-KD, CX3CR1-KD+Vector, CX3CR1-KD+LFA-1 and control A549 cells to validate the effects of CX3CL1/ICAM-1 on NSCLC spinal metastases *in vivo* (Figure 7A).

BLI was conducted every week to examine the development of multiple metastases in mice. As shown in Figure 7B and 7C, genetic inhibition of CX3CR1 in A549 cells led to a roughly 7-day delay in spinal metastasis onset and an 9.2-fold decrease in spinal metastasis burden compared to controls. The metastasis disadvantage was not reversed by transfection of Vector but was significantly rescued by overexpression of LFA-1 in A549 cells with a similar onset of spinal metastasis and a comparable spinal metastasis burden to control mice. Importantly, the survival improvement of CX3CR1-KD -treated mice compared with control mice over a 6-week period was remarkably abolished by overexpression of LFA-1 in NSCLC cells (Figure 7D).

Quantitative microCT analysis of bone regions and decalcified bone sections revealed that CX3CR1 KD in A549 cells significantly reduced the severity of osteolytic bone lesions, with less reduction of osteolytic area and higher relative bone volume than controls (Figure 7E, 7G, and 7H). Histological analysis showed reduced levels of phosphorylated PI3K and AKT, and decreased levels of MMP-2 in CX3CR1-KD groups than those in control groups, indicating downregulation of the aggressive propensity of tumor cells conferred by CX3CR1-KD treatments (Figure 7F). RT-qPCR analyses showed 0.4-0.7-fold decreases in *Icam-1* and *Gef-h1* mRNA levels within spine-metastatic lesions of CX3CR1-KD mice relative to control mice, indicating reduced permeability of VBMECs induced by CX3CR1-KD treatment (Figure 7I). Notably, mice harboring LFA-1-overexpressing NSCLC cells developed more severe spine-metastatic lesions radiographically and higher tumor burdens histologically compared with CX3CR1-KD mice, which was comparable to those in control groups (Figure 7E, 7G, and 7H). These findings demonstrated that overexpression of LFA-1 in the CX3CL1/ICAM-1-mediated cycle could remarkably reverse the inhibition of CX3CR1 KD on NSCLC spinal metastasis. Similar observations were found with phosphorylation levels of PI3K and AKT, protein levels of MMP-2, and mRNA levels of *Icam-1* and *Gef-h1* within spine-metastatic lesions of mice, confirming the rescue effect of LFA-1-overexpression on NSCLC spinal metastases to CX3CR1-KD treatment (Figure 7F and 7I). This evidence suggested that the CX3CL1/ICAM-1-mediated cycle between circulating NSCLC cells and VBMECs accounted for the spinal metastases of NSCLC in a CX3CR1/LFA-1-dependent manner.

To further validate the effects of the CX3CL1/ICAM-1-mediated feedback cycle between circulating NSCLC cells and VBMECs on NSCLC spinal metastases, nude mice were inoculated intracardially with CX3CR1-KD, LFA-1-KD, and CX3CR1-KD and LFA-1-KD A549 cells to disengage the CX3CL1/ICAM-1 interactive signaling network and disrupt tumor-stromal communication (Figure S5A).

BLI was conducted every week to examine the development of multiple metastases in mice. As shown in Figure S5B and S5C, genetic inhibition of CX3CR1 in A549 cells led to a roughly 7-day delay in spinal metastasis onset and an 8.6-fold decrease in spinal metastasis burden compared to controls. A metastasis disadvantage was also induced by silencing of LFA-1 in A549 cells with a 7-day delay in spinal metastasis and a 5.4-fold decrease in spinal metastasis burden. In addition, simultaneous silencing of LFA-1 and CX3CL1 in A549 cells led to a 7-day delay in spinal metastasis and a 9.4-fold decrease in spinal metastasis burden. Importantly, the survival of CX3CR1-KD-, LFA-1-KD-, and CX3CR1-KD-and LFA-1-KD-treated mice significantly improved compared with control mice over a 6-week period (Figure S5D).

Quantitative microCT analysis of vertebral lesions showed less reductions in relative bone volume and smaller osteolytic areas in CX3CR1-KD-, LFA-1-KD-, and CX3CR1-KD- and LFA-1-KD-treated mice than controls (Figure S5E, S5G and S5H). Histological and RT-qPCR analyses showed lower CX3CL1, *CX3CR1*, and *LFA-1* levels in spine-metastatic lesions of the CX3CR1-KD, LFA-1-KD, and CX3CR1-KD and LFA-1-KD groups than those in control groups, indicating disengagement of the CX3CL1/ICAM-1-mediated vicious cycle between circulating NSCLC cells and VBMECs (Figure S5F and S5I). CX3CR1-KD or/and LFA-1-KD treatments also downregulated the aggressive propensity of tumor cells, as evidenced by reduced levels of phosphorylated PI3K and AKT, and decreased levels of MMP-2, compared with controls (Figure S5F). Similar observations were found with *Icam-1* and *Gef-h1* mRNA levels within spine-metastatic lesions of mice injected with CX3CL1‑KD, LFA-1-KD, and CX3CR1-KD and LFA-1-KD NSCLC cells, indicating reduced permeability of VBMECs induced by disengagement of the CX3CL1/ICAM-1-mediated cycle (Figure S5I). This evidence confirmed that the CX3CL1/ICAM-1-mediated cycle between circulating NSCLC cells and VBMECs accounted for the spinal metastases of NSCLC.

## Discussion

In the present study, we investigated the spinal metastasis of NSCLC based on the prominent distribution of CX3CL1/ICAM-1 in vertebral cancellous bone. It was elucidated that expression of CX3CL1 and ICAM-1 was specifically increased in VBMECs and vertebral cancellous bone compared with LBMECs and limb cancellous bone. VBMECs promoted NSCLC cell aggressive capacity via CX3CL1/CX3CR1-dependent activation of the PI3K/AKT pathway while NSCLC cells in turn promoted VBMEC CX3CL1 synthesis through the NF-κB pathway and enhanced CX3CL1 release via the MAPK14/ADMA17 pathway, which created a potent positive feedback loop. Meanwhile, CX3CL1/ICAM-1 molecules enabled circulating NSCLC cell adhesion to VBMECs in cooperation with platelets by activating the CX3CL1/ICAM-1/LFA-1 pathway and drove NSCLC cell transendothelial migration by improving permeability of VBMECs through the Src/GEF-H1 pathway. Our results demonstrated that CX3CL1/ICAM-1 signaling, which underlies tumor cell-endothelium interactions, constituted a vicious feedback cycle between circulating NSCLC cells and the bone microenvironment to promote NSCLC spinal metastasis.

**Figure 8:**
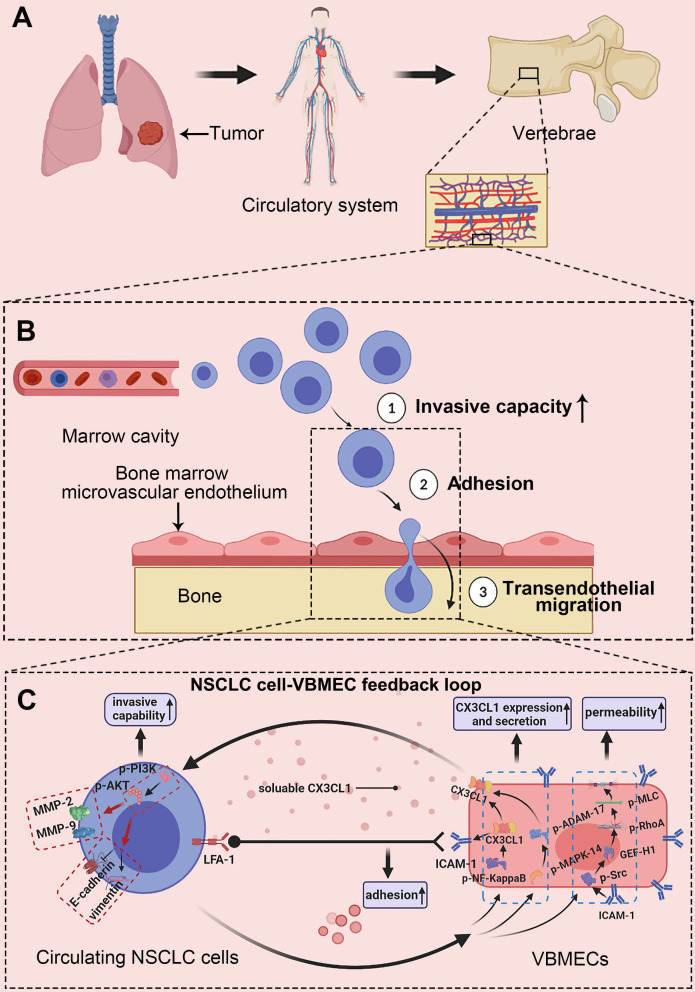
** Schematic depicting the interactions of circulating NSCLC cells with VBMECs mediated by the CX3CL1/ICAM-1 signaling network to induce NSCLC spinal metastasis. (A)** NSCLC metastases to vertebrae via the circulatory system. **(B)** Extravasation of NSCLC cells in cancellous bone. **(C)** The feedback cycle between circulating NSCLC cells and VBMECs based on tumor-endothelium interactions.

Recent studies have suggested that distant metastases of numerous cancers occur in an organ-specific manner, which is dependent on the interaction of cancer cells with the host microenvironment 7. In this study, we reported a CX3CL1/ICAM-1-directed feedback loop between VBMECs and circulating NSCLC cells, which accounted for the spine-specific metastasis of NSCLC. In this feedback loop, VBMECs provided soluble CX3CL1 in the bone microenvironment, which directly acted on circulating NSCLC cells to stimulate their invasion and increase their adhesion; in turn, circulating NSCLC cells stimulated VBMECs to synthesize and secrete CX3CL1 via several relevant signaling pathways, facilitating the increase in VBMEC permeability and constituting the condition-chain of circulating NSCLC cell transendothelial migration (Figure 8). In addition, these results revealed a cellular interaction whereby ICAM-1/LFA-1 promoted adhesion of circulating NSCLC cells to VBMECs, thus increasing their interaction and driving up the functionality of the VBMECs, acting as a multiplier. Therefore, the coupling of CX3CL1-directed cellular interactions and the ICAM-1/LFA-1-mediated feedback loop between circulating NSCLC cells and VBMECs constituted a vicious cycle to recruit more CTCs, eliciting higher vertebral microvascular endothelial permeability and increasing the aggressiveness of tumor cells, which facilitated spinal metastasis of circulating NSCLC cells.

There is emerging evidence that activation of the CX3CL1 signaling pathway in VBMECs plays a crucial role in directing tumor spinal metastasis; this has been supported by primary tumor spinal metastasis in xenografts 13, 14. Our study showed that CX3CL1/ICAM-1 facilitated spinal metastasis of NSCLC in dual-directional ways, namely improving VBMEC permeability and enhancing tumor cell adhesion and invasion in the context of tumor-stromal interactions, revealing three roles of CX3CL1 in directing circulating NSCLC cell extravasation to trigger spinal metastasis. Importantly, CX3CL1 was the central mediator in the interaction between circulating NSCLC cells and VBMECs: the master effector in the ICAM-1-dependant Src/GEF-H1 pathway to regulate VBMEC permeability, the main downstream effector in the NF-κB and MAPK14/ADMA17 pathways for NSCLC cells acting on VBMECs, and a pivotal player in the PI3K/AKT pathway enabling the VBMEC-induced increase of NSCLC cell invasive capacity. Therefore, it is conceivable that the spine-specific increased expression of CX3CL1 was responsible for spinal NSCLC metastases.

ICAM-1 is a single chain transmembrane glycoprotein with a molecular weight of 80-114 kDa, which belongs to the immunoglobulin superfamily of adhesion molecules and is mainly expressed on endothelial cells 38. Evidence suggests that ICAM-1 is involved in cancer progression and promotes distant metastasis of tumors, such as those present in liver cancer, colorectal cancer, and lung cancer 25. Here, we documented that CX3CL1 directed NSCLC cell adhesion to VBMECs depending on ICAM-1 association with LFA-1 in the membrane of NSCLC cells. In addition, CX3CL1 improved VBMEC permeability through ICAM-1-mediated activation of the Src/GEF-H1 pathway. Therefore, we showed that ICAM-1 served as a crucial effector in the dual-directional ways of CX3CL1 to generate interactions with circulating NSCLC cells in NSCLC spinal metastasis and implied that pharmacological inhibition of ICAM-1 signaling may reduce the development of spinal metastasis in clinic.

We reported that platelets played an important role in directing NSCLC cell adhesion to the vascular endothelium. In the context of NSCLC cell-VBMEC interactions, platelets mediated CX3CL1/ICAM-1-dependent cellular association, and thus enhanced the efficacy of the vicious cycle. However, the regulatory mechanisms underlying the stimulatory effect of platelets on NSCLC cell adhesion are still unclear. Recent studies have shown that CTCs can induce platelet aggregation on their surface to form platelet-tumor cell hetero-aggregates, and that adhesion molecules on platelets can further form adhesion bridges with corresponding adhesion molecules on MECs; thus, platelets may act as a bridge in the adhesion between CTCs and the vascular endothelium 39-41. Therefore, we hypothesize that CX3CL1/ICAM-1 and platelets can induce NSCLC cells to adhere to VBMECs alone or in concert. Such a scenario warrants further investigation, as we did not explore the detailed involvement of cellular or molecular interactions among platelets, VBMECs, and CTCs in the process of platelet-assisted NSCLC cell adhesion.

There are two forms of CX3CL1: one present at the cell membrane and the other released outside the cell, which is considered soluble CX3CL1 42. Increasing evidence has demonstrated that CX3CL1 expression is upregulated in tumor metastases 43. In the present study, we found that expression of CX3CL1 was specifically increased in vertebral cancellous bone, and that VBMECs acted as a rich reservoir of CX3CL1, which was released into the bone microenvironment during NSCLC spinal metastasis. Intriguingly, our study revealed the convergence of two functional signaling pathways, namely NF-κB and MAPK14/ADAM17, in the regulation of CX3CL1 secretion when circulating NSCLC cells exerted effects on VBMECs. This means that NSCLC cells interacted with VBMECs to regulate CX3CL1 production in two ways: by promoting CX3CL1 gene expression via the NF-κB pathway and by enhancing soluble CX3CL1 secretion via MAPK14/ADAM17 signaling. ADAM17 has been shown to be involved in the release of more than 70 kinds of transmembrane cytokines, growth factors, and cell surface receptors 44, 45. In this study, we found that NSCLC cells stimulated VBMECs to release soluble CX3CL1 primarily through cleavage of the extracellular domain of CX3CL1 by proteolytically active ADAM17. Additionally, considering that CX3CL1 induced more circulating NSCLC cells to contribute to CTC-VBMEC interactions by promoting their adhesion to VBMECs, it is conceivable that CX3CL1 also promoted itself production by driving positive feedback during the vicious cycle in response to circulating NSCLC cell stimulation.

In the present study, CX3CL1/ICAM-1 promoted NSCLC cell transendothelial migration via upregulation of VBMEC permeability. However, the regulatory mechanisms have not been thoroughly investigated. The present study detected the expression of Src, GEF-H1, RhoA, and ROCK1, and demonstrated that CX3CL1/ICAM-1 upregulated GEF-H1 and ROCK1 expression as well as promoted RhoA phosphorylation via activation of Src. GEFs regulate the GDP/GTP transformation of Rho family GTPases, which has been shown to regulate cytoskeleton remodeling and endothelial permeability by regulating the kinase activity of RhoA 46. RhoA signaling and its downstream effectors, which include cleaved ROCK1 and phosphorylated MLC, are known to regulate cell migration through reorganization of the cytoskeleton 28, 47. Therefore, we hypothesize that CX3CL1/ICAM-1 induces activation of RhoA through the Src/GEF-H1 pathway to increase MLC phosphorylation, thus promoting actin-myosin contraction and increasing the permeability of VBMECs.

Previous researches found that CX3CL1/CX3CR1 axis was associated with the survival, adhesion, and migration of malignant tumor cells, including hepatocellular carcinoma, prostate cancer, and breast cancer 48, 49. In the present study, we found that VBMECs effectively induced NSCLC cell adhesion in coordination with platelets through the CX3CL1/ICAM-1/LFA-1 pathway, and that CX3CL1/ICAM-1 signaling remarkably increased VBMEC permeability via activating the Src/GEF-H1 pathway. Thus, CX3CL1/ICAM-1 signaling plays a vital role in the novel feedback cycle between circulating NSCLC cells and VBMECs. However, CX3CL1/CX3CR1 and ICAM/LFA may work independently from one another. Liu *et al.* demonstrated that CX3CL1/CX3CR1 enhanced prostate cancer spinal metastasis by activating the Src/FAK pathway. Cambien *et al.* reported that CX3CL1/CX3CR1 axis induced phosphorylation of PI3K, Erk1/2, Akt, p38, and Src 50. Consistently, we also found that VBMECs enhanced NSCLC cell invasion via CX3CL1/CX3CR1 signaling-mediated activation of PI3K/AKT pathway. Additionally, previous studies reported that ICAM expression and the activation of ICAM/LFA signaling pathway could be upregulated by interferon-γ, IL-1β, and TNFα, and the increased expression of ICAM/LFA has been shown to facilitate tumor metastasis and progression 51, 52. Therefore, we speculated that CX3CL1/CX3CR1 and ICAM/LFA, may as well work independently from one another.

In summary, our results showed that highly expressed CX3CL1/ICAM-1 in vertebrae facilitated NSCLC spinal metastasis by driving a spine-specific vicious cycle between circulating NSCLC cells and VBMECs. This cycle is based on CX3CL1/ICAM-1-mediated NSCLC cell-VBMEC interactions resulting in enhanced tumor cell aggressiveness and adhesion, improved VBMEC permeability, and increased NSCLC cell transendothelial migration through several signaling pathways. The mechanisms described in the present study that underlie NSCLC spinal metastasis may provide potential novel targets for the prevention of NSCLC spinal metastasis.

## Supplementary Material

Supplementary figures.

## Figures and Tables

**Table 1:** Antibodies used in this study.

Antibody	Species	Target Species	Dilution Ratio	Supplier	Catelogue Number
CX3CL1	Rbt	H, M, R	1:1000	Abcam	ab25088
CX3CR1	Rbt	H, M, R	1:1000	Abcam	ab8021
ICAM-1	Rbt	H	1:1000	Abcam	ab53013
LFA-1	Rbt	H	1:1000	CST	no. 73663
Src	Rbt	H, M, R	1:1000	CST	no. 2108
p-Src (Tyr416)	Rbt	H, M, R	1:1000	CST	no. 6943
Akt	Rbt	H, M, R	1:1000	CST	no. 4691
p-Akt (Ser473)	Rbt	H, M, R	1:1000	CST	no. 4060
PI3K	Rbt	H, M, R	1:1000	CST	no. 4249
p-PI3K (Ser249)	Rbt	H	1:1000	CST	no. 13857
Vimentin	Rbt	H, M, R	1:1000	CST	no. 5741
E-cadherin	Rbt	H, M	1:1000	CST	no. 3195
MMP-2	Rbt	H	1:1000	CST	no. 13132
MMP-9	M	H, M, R	1:1000	SC	sc-393859
GEF-H1	Rbt	H, M, R	1:1000	CST	no. 4076
ROCK1	Rbt	H, M, R	1:1000	CST	no. 4035
RhoA	Rbt	H, M, R	1:1000	Abcam	ab187027
p-RhoA (Ser188)	Rbt	H	1:1000	Abcam	ab41435
MLC	Rbt	H, M, R	1:1000	CST	no. 3672
p-MLC (Ser19)	Rbt	H, M, R	1:1000	CST	no. 3671
MAPK14	M	H, M, R	1:1000	Abcam	ab31828
p‑MAPK14 (Tyr180)	Rbt	H	1:1000	Abcam	ab4822
ADAM17	Rbt	H, M, R	1:1000	Abcam	ab2051
p‑ADAM17(Tyr735)	Rbt	H, M, R	1:1000	Abcam	ab182630
NF‑κB	Rbt	H, M, R	1:1000	Abcam	ab16502
p-NF‑κB (Ser536)	Rbt	H, M, R	1:1000	CST	no. 3033
β‑actin	M	H, M, R	1:1000	CST	no. 3700
GAPDH	Rbt	H, M, R	1:1000	Beyotime	no. AF1186
Anti-Rabbit IgG	Goat	Rbt	1:5000	YEASEN	no. 33101ES60
Anti-Mouse IgG	Goat	M	1:5000	YEASEN	no. 33201ES60

Abbreviations: H, human; Rbt, rabbit; R, rat; M, mouse; CST, Cell Signaling Technology (Danvers, MA, USA); SC, SANTA CRUZ Biotechnology (Dallas, Texas, USA); Beyotime, Beyotime Institute of Biotechnology (Shanghai, China); YEASEN, YEASEN Biotech (Shanghai, China); R&D, R&D SYSTEMS (Minneapolis, MN, USA).

**Table 2:** Primer sequences for qPCR.

Genes	Primer sequences	Annealing temperature
*CX3CL1*	forward 5'-GACCCCTAAGGCTGAGGAAC-3'	60 ℃
	reverse 5'-AGAAGAGGAGGCCAAGGAAG-3'	
*ICAM-1*	forward 5'-AGCGGCTGACGTGTGCAGTAAT-3'	
	reverse 5'-TCTGAGACCTCTGGCTTCGTCA-3'	
*CX3CR1*	forward 5'-GACGGTTGCATTTAGCCATT-3'	
	reverse 5'-TGCTCAGAACACTTCCATGC-3'	
*ADAM17*	forward 5'‑GTGAGCAGTTTCT CGAACGC‑3'	
	reverse 5'‑AGCTTCTCAAGTCGCAGGTG‑3'	
*GEF-H1*	forward 5′-CCGAGACAAACGCTTCCAGCAA-3′	
	reverse 5′-GGTACTTGGTGATGCGCTGAGT-3′	
*LFA-1*	forward 5′-TGCGTCCTCTCTCAGGAGTG-3′	
	reverse 5′-GGTCCATGATGTCGTCAGCC-3′	

*CX3CL1*, C‑X3‑C motif chemokine ligand 1; *ICAM-1*, intercellular adhesion molecule-1; *CX3CR1*, C‑X3‑C motif chemokine receptor 1; *ADAM17*, a disintegrin and metalloproteinase 17; *GEF-H1*, Rho guanine nucleotide exchange factors-H1; *LFA-1*, lymphocyte function-associated antigen-1.

## Abbreviations

NSCLC: non-small cell lung cancer
CX3CL1: C-X3-C motif chemokine ligand 1
ICAM-1: intercellular adhesion molecule-1
RT-qPCR: reverse transcription‑quantitative polymerase chain reaction
microCT: micro-computerized tomography
BLI: bioluminescent
VBMECs: vertebral bone marrow endothelial cells
LBMECs: limb bone marrow endothelial cells
CTCs: circulatory tumor cells
DMEM: Dulbecco's modified Eagle's medium
LFA-1: lymphocyte function-associated antigen-1
ELISA: Enzyme-linked immunosorbent assay
IgG: immunoglobulin
EMT): epithelial-to-mesenchymal transition
MMPs: matrix metalloproteinases
NF-κB: Nuclear factor kappa B
RhoA: Ras Homolog Family Member A
MLC: myosin light chain
GEF: Rho guanine nucleotide exchange factor
ROCK1: Rho-associated kinase 1
