# Supplementary material for: Vertebral-specific activation of the CX3CL1/ICAM-1 signaling network mediates non-small-cell lung cancer spinal metastasis by engaging tumor cell-vertebral bone marrow endothelial cell interactions
Source: Theranostics. 2021 Mar 4;11(10):4770–89. doi: 10.7150/thno.54235 (PMC7978319; doi:10.7150/thno.54235)
Supplement: Supplementary file 1 — Supplementary figures. [file thnov11p4770s1.pdf]

Supplementary material

Vertebral-specific activation of the CX3CL1/ICAM-1 signaling network mediates non-small-cell lung cancer spinal metastasis by engaging tumor cell-vertebral bone marrow endothelial cell interactions

Ketao Wang[1]*, Libo Jiang[1]*, Annan Hu[1]*, Chi Sun[1], Lei Zhou[1], Yiwei Huang[4], Qing Chen[1], Yiqun Ma[1], Jian Dong[1,3], Xiaogang Zhou[1], and Feng Zhang[1,2]

1. Department of Orthopaedic Surgery, Zhongshan Hospital, Fudan University, Shanghai 200032, China;
2. JMS Burn and Reconstruction Center, Jackson, Mississippi, USA;
3. Department of Orthopaedic Surgery, Zhongshan Hospital Wusong Branch, Fudan University, Shanghai 200940, China;
4. Department of Thoracic Surgery, Zhongshan Hospital, Fudan University, Shanghai 200032, China.

Corresponding author: Jian Dong, Department of Orthopaedic Surgery, Zhongshan Hospital, Fudan University, 180 Fenglin Road, Shanghai 200032, China. E-mail: dong.jian@zs-hospital.sh.cn; Xiaogang Zhou, Department of Orthopaedic Surgery, Zhongshan Hospital, Fudan University, 180 Fenglin Road, Shanghai 200032, China. E-mail: zhou.xiaogang@zs-hospital.sh.cn; Feng Zhang, JMS Burn and Reconstruction Center, Jackson, Mississippi, USA. E-mail: feng.zhang@burncenters.com.

* Ketao Wang, Libo Jiang and Annan Hu contributed equally to this work.

Supplementary data

Figure S1

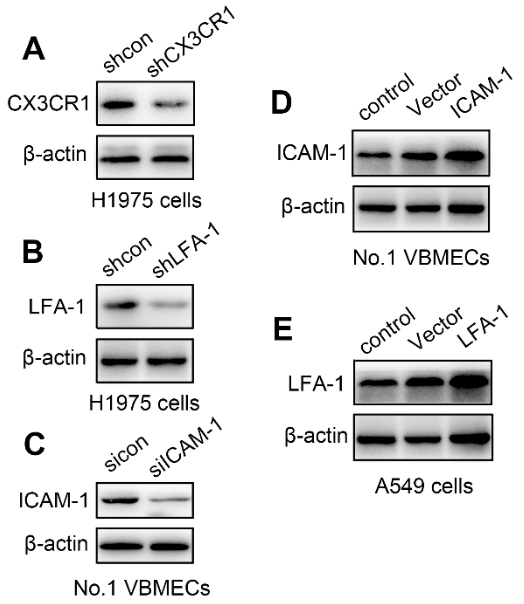

Figure S1. Generation of various cell lines. (A) Reduced protein levels of CX3CR1 in shCX3CR1-H1975 cells. **(B)** Reduced protein levels of LFA-1 in shLFA-1-H1975 cells. **(C)** Reduced protein levels of ICAM-1 in siICAM-1-VBMECs. **(D)** Western blot analysis of ICAM-1 protein levels in ICAM-1-overexpressing VBMECs. **(E)** Western blot analysis of LFA-1 protein levels in LFA-1-overexpressing A549 cells.

Figure S2

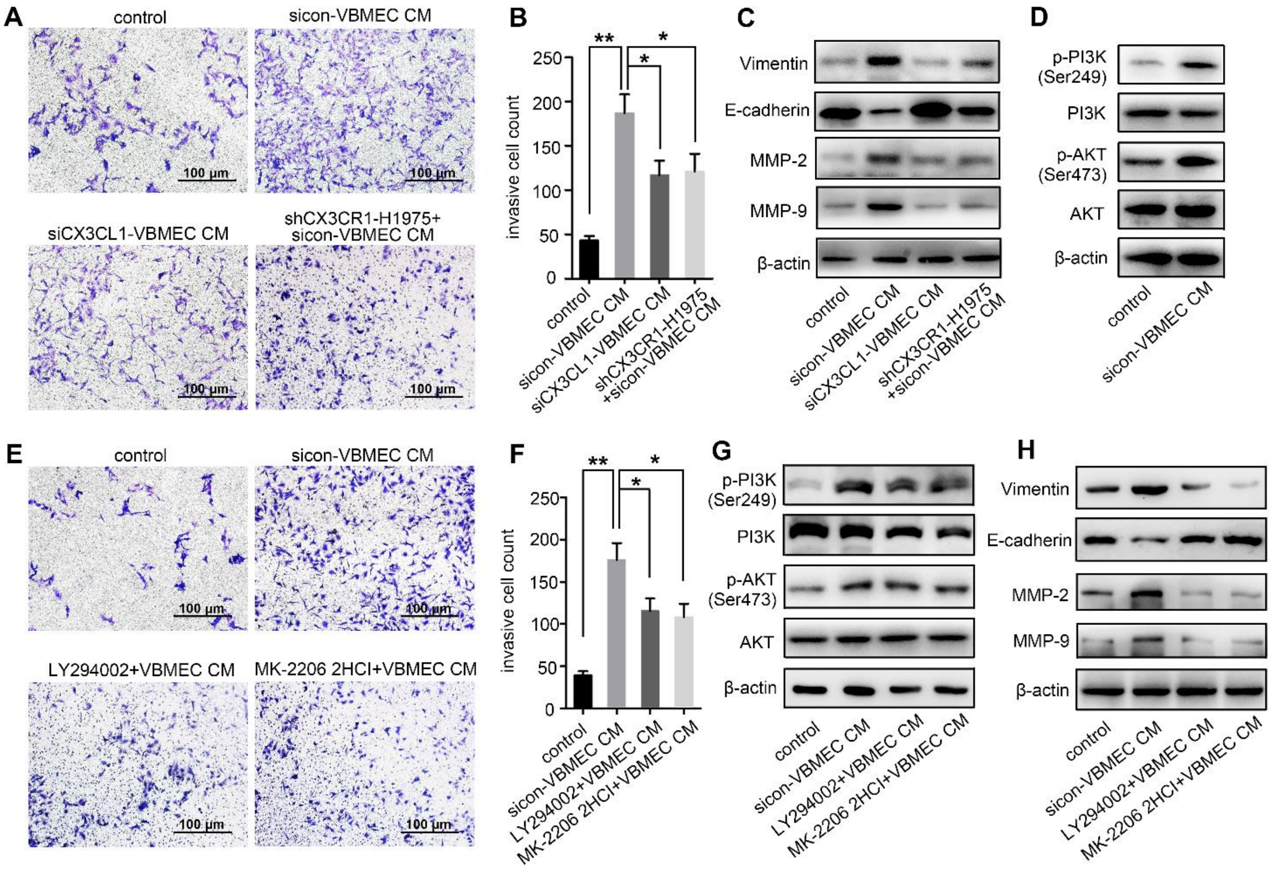

Figure S2. VBMECs enhanced NSCLC cell invasion via CX3CL1 signaling-mediated activation of the PI3K/AKT pathway. (A, B) Quantitative analysis of cell invasion in control (shcon) and CX3CR1-KD (shCX3CR1) H1975 cells incubated with or without conditioned culture media of indicated VBMECs (VBMEC CM). Data represent the mean ± SEM (n = 3). *P < 0.05 and **P < 0.01. **(C)** Expression levels of EMT markers including Vimentin, E-cadherin, MMP-2, and MMP-9 in control (shcon) and CX3CR1-KD (shCX3CR1) H1975 cells co-cultured with or without indicated VBMEC conditioned media measured by western blotting. **(D)** PI3K and AKT phosphorylation in H1975 cells co-cultured with or without VBMEC conditioned media measured by western blotting. **(E, F)** Quantitative analysis of invasion of H1975 cells incubated with or without conditioned culture media of VBMECs treated with the PI3K inhibitor LY294002 or AKT inhibitor MK-2206 2HCl. Data represent

the mean ± SEM (n = 3). $*P < 0.05$ and $**P < 0.01$. **(G, H)** Expression levels of PI3K, p-PI3K (Ser249), AKT, p-AKT (Ser473), Vimentin, E-cadherin, MMP-2, and MMP-9 in H1975 cells co-cultured with indicated VBMECs and treated with LY294002 or MK-2206 2HCl measured by western blotting.

Figure S3

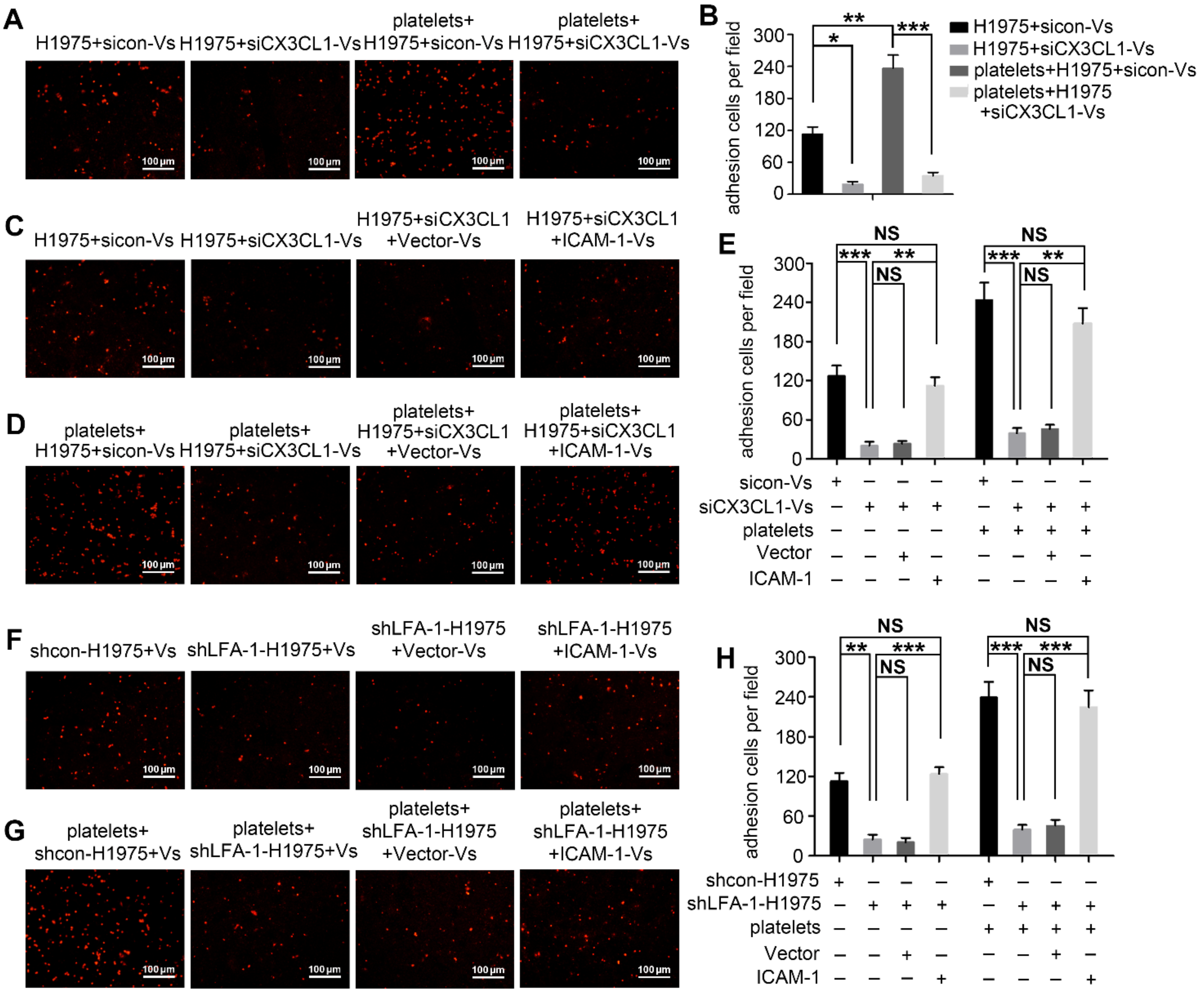

Figure S3. VBMECs induced NSCLC cell adhesion through the CX3CL1/ICAM-1/LFA-1 pathway in the presence or absence of platelets. (A, B) Quantitative analysis of H1975 cell adhesion to indicated VBMECs in the presence or absence of platelets. Data represent the mean ± SEM (*n* = 3). **P* < 0.05, ***P* < 0.01, and ****P* < 0.001. **(C–E)** Overexpression of ICAM-1 in VBMECs significantly rescued the efficacy of CX3CL1 silencing to decrease NSCLC cell adhesion to VBMECs. Data represent the mean ± SEM (*n* = 3). ***P* < 0.01, ****P* < 0.001, and $^{NS}P > 0.05$. **(F–H)** Overexpression of ICAM-1 in VBMECs significantly reversed the decrease in NSCLC cell adhesion to VBMECs induced by LFA-1-KD treatment. Data represent the mean ± SEM (*n* = 3). ***P* < 0.01, ****P* < 0.001, and $^{NS}P > 0.05$.

Figure S4

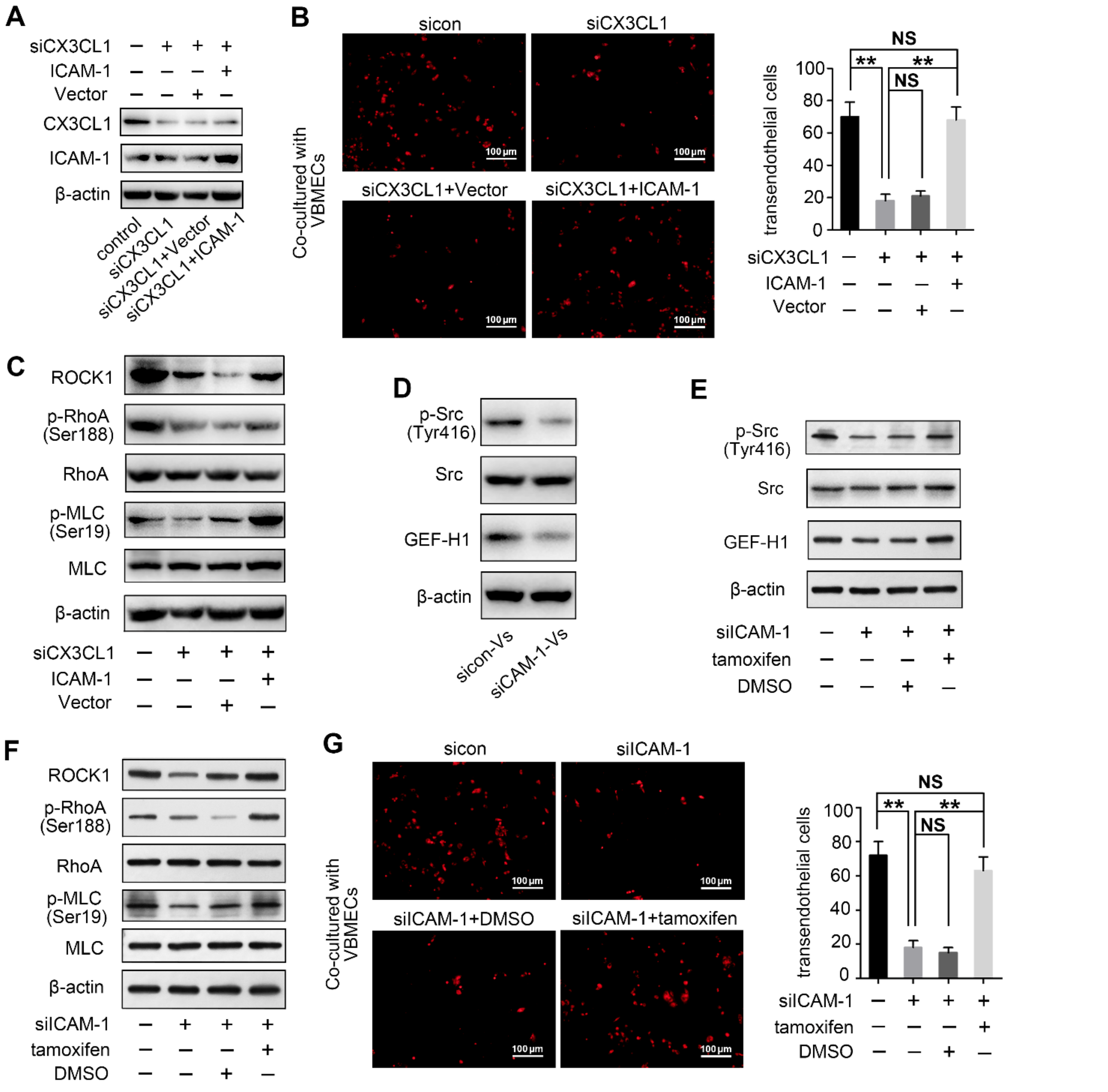

Figure S4. CX3CL1/ICAM-1 enhanced NSCLC cell transendothelial migration by increasing VBMEC permeability through the Src/GEF-H1 pathway. (A) Western blot analysis of CX3CL1 and ICAM-1 protein levels in indicated VBMECs. **(B)** Quantitative analysis of H1975 cell transendothelial migration with control (sicon), CX3CL1-KD (siCX3CL1), CX3CL1-KD and Vector (siCX3CL1+Vector), or CX3CL1-KD and ICAM-1-overexpression (siCX3CL1+ICAM-1) VBMECs as an endothelial monolayer barrier. Data represent the mean ± SEM (n = 3). $^{**}P < 0.01$, and $^{NS}P > 0.05$. **(C)** Western blotting of RhoA, p-RhoA, MLC, p-MLC, and ROCK1 protein levels in the indicated

VBMECs treated with conditioned media from H1975 cells. **(D)** Src, p-Src, and GEF-H1 levels in the indicated VBMECs treated with conditioned media from H1975 cells detected by Western blot analysis. **(D)** Src, p-Src, and GEF-H1 levels in the indicated VBMECs incubated with conditioned media from H1975 cells, treated with the Src signaling pathway agonist tamoxifen. **(F)** Western blotting of RhoA, p-RhoA, MLC, p-MLC, and ROCK1 protein levels in the indicated VBMECs incubated with conditioned media from H1975 cells, treated with tamoxifen. **(G)** Quantitative analysis of NSCLC cell transendothelial migration with administration of tamoxifen in the indicated co-cultures of VBMECs and H1975 cells. Data represent the mean ± SEM (n = 3). ***P < 0.001, **P < 0.01, and ^{NS}P > 0.05.

Figure S5

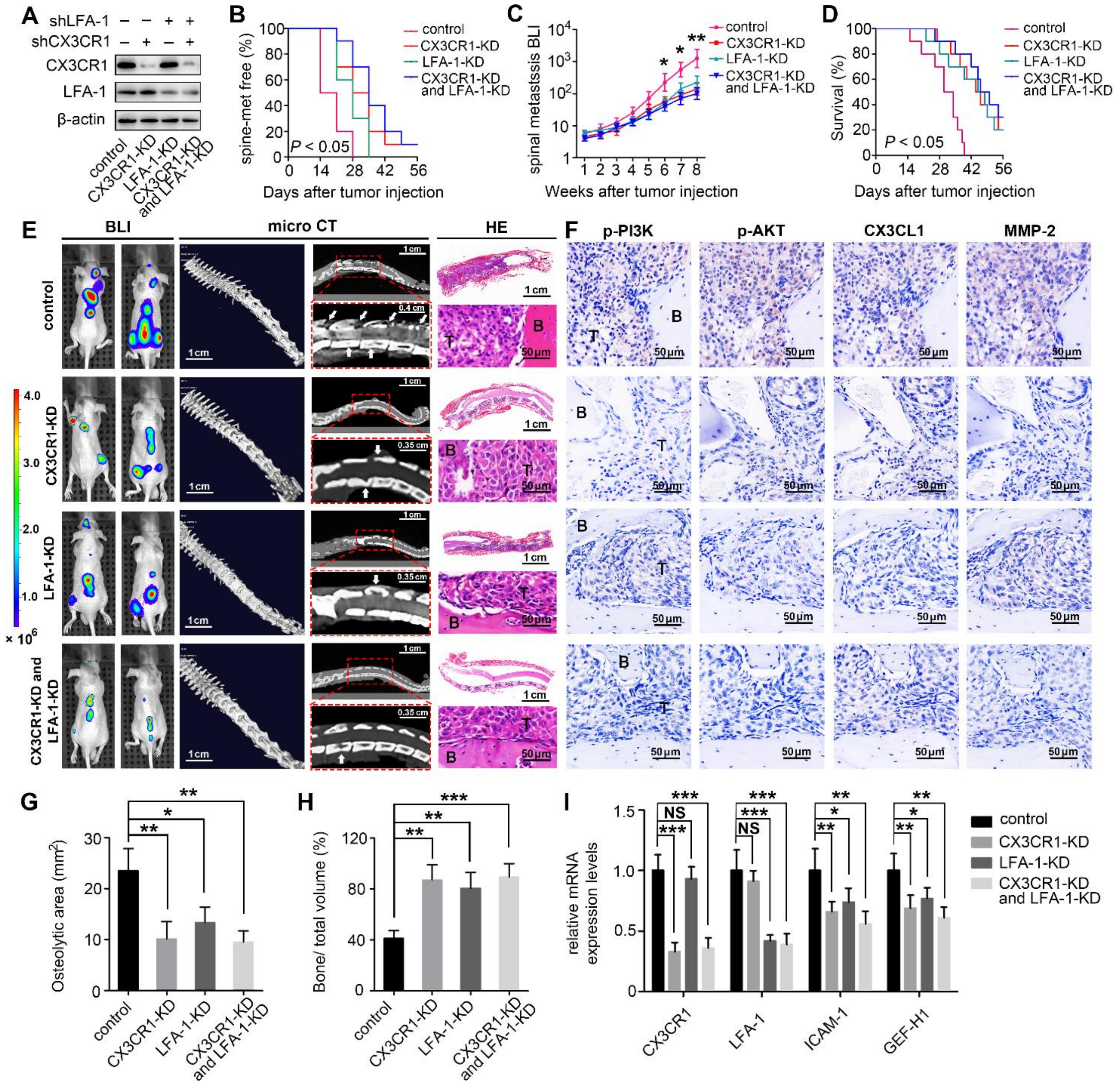

Figure S5. Disengagement of the CX3CL1/ICAM-1-mediated feedback cycle between circulating NSCLC cells and VBMECs by silencing CX3CR1 and/or LFA-1 in A549 cells reduced NSCLC spinal metastasis and prolonged survival in mice. (A) Western blotting of CX3CR1 and LFA-1 protein levels in the indicated A549 cells. **(B)** Kaplan-Meier spinal metastasis-free curve of mice inoculated intracardially with CX3CR1-KD, LFA-1-KD, or CX3CR1-KD and LFA-1-KD A549 cells (n = 10). **(C)** Normalized BLI signals of spinal metastases. Data represent the mean ±SD (n = 10). *P < 0.05 and **P < 0.01. **(D)** Kaplan–Meier survival curve of mice. **(E)** Representative

BLI, microCT, and histological (HE) images of bone lesions (B) invaded by tumors (T) in each group. Arrows indicate osteolytic bone lesions and dashed rectangles are used to mark the regions of interest for vertebral bone scan and analysis. **(F)** IHC images of p-PI3K, p-AKT, CX3CL1, and MMP-2, in tumor areas from serial sections of indicated samples. **(G, H)** Quantification of osteolytic areas of spine and bone volume relative to total volume from microCT scans. Data represent the mean ± SEM (n = 5). *P < 0.05, **P < 0.01, and ***P < 0.001. **(I)** mRNA levels of *CX3CR1*, *LFA-1*, *Icam-1*, and *Gef-h1* in tumors of mice by RT-qPCR. Data represent the mean ± SEM (n = 10). *P < 0.05, **P < 0.01, ***P < 0.001, and ^{NS}P > 0.05.
